# Adoption of Internet of Things in Health Care: Weighted and Meta-Analytical Review of Theoretical Frameworks and Predictors

**DOI:** 10.2196/64091

**Published:** 2026-01-06

**Authors:** Inês Veiga, Tiago Oliveira, Mijail Naranjo-Zolotov, Ricardo Martins, Stylianos Karatzas

**Affiliations:** 1 NOVA Information Management School (NOVA IMS) Lisbon Portugal; 2 Department of Product & Systems Design Engineering University of the Aegean Hermópolis Greece

**Keywords:** IoT health care, internet of things, individual adoption, weight-analysis, meta-analysis

## Abstract

**Background:**

The integration of the Internet of Things (IoT) into health care is transforming the industry by enhancing disease care and management, as well as supporting self-health management. The COVID-19 pandemic has accelerated the adoption of IoT devices, particularly wearable medical devices, which enable real-time health monitoring and advanced remote health management. Globally, the increased adoption of IoT in health care has improved efficiency, enhanced patient care, and generated substantial economic value.

**Objective:**

This review aims to conduct a comprehensive meta- and weight analysis of quantitative studies to identify the most influential predictors and theoretical frameworks explaining the adoption of IoT in health care.

**Methods:**

We searched databases, including Web of Science and PubMed, for quantitative studies on IoT health care adoption, with the last search conducted in early July 2025. Inclusion criteria comprised peer-reviewed articles written in English that employed a quantitative approach to IoT health care technology adoption. Studies were excluded if they did not report the significance of relationships, involved technologies without IoT features or were outside the scope, or examined target variables irrelevant to the analysis. The weight analysis identified the pathways with the most significant effects. A meta-analysis using a random-effects model was conducted to estimate combined effect sizes and their statistical significance. The results from both methods were then integrated to visualize the most frequently used theoretical frameworks. Risk of bias and heterogeneity were assessed using a funnel plot, Egger regression test, the I2 statistic, and subgroup analysis, which indicated no strong evidence of publication bias but revealed a high level of heterogeneity.

**Results:**

Analysis of 115 datasets from 109 papers identified the Technology Acceptance Model and the Unified Theory of Acceptance and Use of Technology (UTAUT) as the primary frameworks for explaining IoT adoption in health care. Incorporating context-specific variables—such as health consciousness, innovativeness, and trust—into these traditional technology acceptance frameworks enhances the understanding of IoT adoption. Although high heterogeneity suggests a need to refine theoretical models to account for regional contexts, universal adoption drivers such as performance expectancy and effort expectancy remain consistent.

**Conclusions:**

Behavioral intention is the most frequently studied variable in IoT health care adoption, whereas attitude, performance expectancy, effort expectancy, and task-technology fit remain underexplored. While adoption theories from the information systems field, such as the TAM, are predominantly used, integrating context-specific constructs and theories—such as trust and innovativeness—can provide deeper insights into IoT adoption in health care. The strongest and most consistent predictors of behavioral intention were attitude, performance expectancy, habit, self-efficacy, functional congruence, and benefits. Additionally, social influence, facilitating conditions, trust, and aesthetic appeal demonstrated promising or strong effects. By contrast, variables such as privacy and security, barriers, vulnerability, severity, compatibility, financial cost, health, and technology anxiety were generally inconsistent or not statistically significant.

## Introduction

The integration of the Internet of Things (IoT) into health care has revolutionized the industry by introducing a new paradigm of connectivity and data exchange, driven by rapid advancements in IoT, artificial intelligence, and machine learning [1-3]. This era, known as Healthcare 4.0, can be leveraged to enhance acute disease care, manage chronic diseases, and support self-health management [4]. The COVID-19 pandemic accelerated the adoption of user-friendly IoT devices [5-7], with wearable medical devices emerging as key allies by offering real-time health monitoring, continuous data transmission, and advanced remote health management [8-10].

Globally, the integration of IoT in health care has enhanced efficiency, improved patient care, and generated significant economic value [11,12]. By 2029, the global IoT health care market volume is projected to reach US $134.40 billion [13]. This indicates strong, sustained growth driven by the increasing adoption of IoT technologies in health care around the globe [13,14]. For instance, China has made significant progress in integrating health information technologies into the health care system, driven by initiatives such as “Internet Plus Health Care” and the “Healthy China 2030” plan [15-17]. The United States leads in IoT and intelligent health care system development, supported by substantial investments and a robust ecosystem of startups and tech companies driving advancements in artificial intelligence and IoT [18-20]. Europe also shows considerable progress, emphasizing regulatory frameworks, standardization, and interoperability to foster innovation and data protection [21,22].

While IoT holds considerable promise to transform health care by reducing costs and improving access, understanding the factors influencing its adoption requires more focused research [23]. Although literature reviews with a quantitative approach have examined technology adoption in health care, existing meta-analyses that include technologies with IoT features remain fragmented, as they have largely focused on broader or adjacent technological domains and have typically emphasized a specific adoption model. For instance, meta-analyses on mobile health have focused on the Unified Theory of Acceptance and Use of Technology (UTAUT) [24] and the Technology Acceptance Model (TAM) [25]. Meta-analyses on eHealth have predominantly focused on the TAM [26] and continuance intention [27]. Meta-analyses specific to smart wearable health care devices have examined attitude and intention using UTAUT and TAM [28], as well as the effects of perceived usefulness and perceived ease of use on intention, with a focus on Hofstede’s cultural dimensions as moderators [29]. Taken together, these studies often treat health care technology adoption in general terms and do not account for the unique characteristics of IoT.

Our study addresses this gap by providing a comprehensive meta-analysis and a weight analysis specifically focused on IoT adoption in health care. We synthesize findings from primarily quantitative articles on the adoption of IoT in health care, particularly on interconnected devices that monitor and transmit real-time health care data, enabling smarter solutions [5], such as smart sensors, remote monitoring devices, and health-focused IoT platforms. Our meta-analytic approach integrates findings from different theoretical perspectives, including technology adoption models such as the TAM and UTAUT and health-specific models such as the Health Belief Model (HBM), allowing for a more holistic understanding of adoption dynamics in health care contexts. Moreover, our dual-method approach, combining meta-analysis with weight analysis, identifies the strongest and most reliable predictors of adoption and maps the theoretical foundations most frequently and effectively used in this field. This analysis goes beyond prior reviews, offering new evidence-based insights to guide health care technology developers, practitioners, and researchers. The objectives of this study are, first, to identify key predictors of IoT health care adoption through a comprehensive meta-analysis and weight analysis, and second, to determine the most influential and empirically supported theories used to explain IoT adoption in health care settings.

## Methods

### Overview

We performed a meta-analysis to examine the factors influencing the adoption of IoT technologies in health care, synthesizing findings from a range of quantitative studies. By focusing on primary quantitative research articles, we aimed to identify the most significant predictors and the theoretical models most commonly used to explain IoT adoption in health care settings.

### Information Sources and Search Strategy

This meta-analysis followed PRISMA (Preferred Reporting Items for Systematic Reviews and Meta-Analyses) 2020 guidelines [30]. The literature search was conducted using a keyword-based search across the Web of Science and PubMed databases to identify studies examining IoT adoption in health care. The search strategy incorporated title, abstract, and keyword searches, using Boolean operators (AND and OR) and database-specific filters. The keywords used in our search were related to IoT technology, relevant variables, quantitative methods, and exclusion criteria (Table 1). We included records published up to the end of 2024. The complete search strategy, including search terms and Boolean logic, is provided in Multimedia Appendix 1.

**Table 1:** Map for the keyword search in online databases.

Relevant terms	Relevant variables and theories	Methodologies	Exclusion of irrelevant topics
Internet of Things	Intention to adopt	Structural equation	Systematic
IoT	Behavioral intention	Structural equation modeling	Literature review
Smart	Acceptance	Partial least squares structural equation modeling	Postadoption
Intelligent	Adopt	Partial least	Meditation
Health care wearable device	Adoption	Path analysis	Contact tracing app
Medical wearable technology	Using	Regression	Fitness app
Health management	Use	N/A^a^	Electronic health record
Health measurement	Usage	N/A	Telemedicine
N/A	Intention to use	N/A	Mindfulness app
N/A	Unified Theory of Acceptance and Use of Technology 2	N/A	N/A
N/A	Unified Theory of Acceptance and Use of Technology	N/A	N/A
N/A	Technology Acceptance Model	N/A	N/A

^a^N/A: not applicable.

### Selection Criteria

Initial screening was conducted using database filters. In the second screening, 2 (IV and MNZ) independent reviewers assessed the titles and abstracts for relevance, resolving disagreements through discussion or arbitration by a third reviewer. The full-text screening followed the same procedure. The reports assessed for eligibility were exported to an Excel (Microsoft Corporation) file, and all included papers were imported into Zotero (Corporation for Digital Scholarship), which is a reference management software. When a paper was unavailable, the authors were contacted.

Inclusion criteria comprised peer-reviewed articles with a quantitative approach to health care technology adoption written in English. Reasons for exclusion included not reporting the significance of the relationships between variables; the technology lacking IoT features or being unrelated to health care; the target variables being unrelated to adoption or focusing solely on postadoption behaviors; and studies lacking empirical data or reporting qualitative results only. The workflow and search conditions are depicted in more detail in the “Results” section.

### Data Extraction

A standardized data extraction form was developed before data extraction. Data extraction was performed in Excel, and for each article, we detailed the study characteristics, methodology, type of technology, and the effects measured across multiple paths. The extracted aspects and their descriptions are provided in Table S1 in Multimedia Appendix 1. We assessed paper quality by examining the publishing journal metrics, the methods employed, sample size, and the scales used to measure each construct. The standardized β coefficients were extracted as the primary effect measure. As some authors used different names to represent the same variable, several variables had to be merged to conduct our analysis. This process was carried out by reading each variable definition and identifying the items used to measure them. Examples of variable mergers are provided in Table S2 in Multimedia Appendix 1, and the individual studies included in the analysis are detailed in Table S3 in Multimedia Appendix 1.

### Descriptive Analysis

We extracted metadata from each study to perform a descriptive analysis of publication trends, journal quality, and research domains. Data on publication year were used to assess the chronological distribution of studies. To evaluate journal quality, we matched each journal with its SCImago Journal & Country Rank classification and categorized them into quartiles (Q1-Q4). The disciplinary scope of the journals was identified based on their SCImago subject area classifications. We also recorded the journal title and publication frequency to identify the journals that published the most research. Country-level data were extracted based on the origin of the study sample or study location. We computed the number of studies and total sample sizes per country to identify regions with the highest research activity. To understand the theoretical foundations employed across studies, we reviewed each article’s methodology and coded the theories used to model technology adoption behavior. We also recorded whether these models were used independently or in combination (eg, UTAUT extended with Protection Motivation Theory [PMT] constructs).

### Weight Analysis

The weight analysis was conducted to uncover the predictive power of independent variables [31]. This weight provides a measure of the relative importance or consistency of statistical significance for each variable across multiple analyses. For the weight-analytic approach, we focused on the influence of each independent variable on several dependent variables and limited our analysis to relationships investigated 3 or more times [32,33]. The weight (*W_i_*) of an independent variable *i* is calculated as the ratio of the number of times it was found to be statistically significant (*S_i_*) to the total number of times it was examined (*E_i_*), as expressed in the following equation:

*W_i_* = *S_i_*/*E_i_*

### Meta-Analysis

Meta-analyses allow us to quantitatively compare effect sizes across relationships between constructs using suitable metrics to capture these effect sizes, including standardized regression coefficients [34,35]. This analysis followed best practices outlined previously [36-38]. In our study, the necessary inputs for performing the meta-analysis were the standardized regression coefficients (*β*) and the sample sizes for each relationship examined 3 or more times across studies. Following the approach of Peterson and Brown [37], *β* values were transformed into approximate correlation coefficients as *r*=*β*+0.05, where λ=1 [37]. All correlation coefficients were Fisher *z*-transformed to stabilize variance, and SEs were computed.

A random-effects model was used to account for both within- and between-study variance, justified by the heterogeneity in study populations, methods, and contexts. Random-effects weights were calculated using the DerSimonian and Laird model, and weighted mean effect sizes were computed using inverse-variance weights, which use tau-squared (τ^2^) [39,40]. Heterogeneity was assessed using the *Q* statistic and the *I*^2^ index [41]. We also calculated the lower and upper bounds of the 95% CIs, *z* scores, and 2-tailed *P* values to assess statistical significance and interpret the magnitude of the observed effects. Final pooled effect sizes and CIs were then back-transformed from Fisher *z* to the correlation coefficient metric (*r*). All calculations were performed manually in Excel.

### Publication Bias Analysis

The Egger test was used to statistically examine the presence of publication bias by regressing the standard normal deviate on precision [42]. The analysis was performed using Excel’s data analysis regression tool, which applies standard ordinary least squares regression, and a significant intercept (*P*<.10) was interpreted as evidence of asymmetry and possible publication bias. A funnel plot was constructed to visually assess publication bias using the tool Meta-Essentials [43]. The trim-and-fill method was used to estimate the number and influence of missing studies. Heterogeneity for the included studies was assessed using the *I*^2^ statistic, where a value over 75% is interpreted as substantial heterogeneity, using the following formula, where *k* is the number of studies and *Q* the Cochran *Q* statistic:

*I*^2^ = max(0; {*Q* – [*k* – 1]}/*Q*) × 100%

To evaluate regional bias, we conducted a subgroup analysis with 2 groups: one comprising studies conducted in China and the other comprising studies conducted in the remaining countries. For each group, we calculated the combined effect sizes, SEs, CI lower and upper limits, and the *I*^2^ statistic.

### Combining Weight and Meta-Analysis Results: The Most Used Adoption Models

To synthesize the relative strength of relationships across studies and adoption models, we combined the results from the weight analysis and meta-analysis. The weight analysis assessed the consistency and prominence of specific predictors by calculating the proportion of studies that reported statistically significant relationships for each path, referred to as the weight. In parallel, the meta-analysis provided pooled average effect sizes and significance levels across studies using a random-effects model. This dual approach offers a more comprehensive understanding of which constructs consistently predict behavioral intention or usage in the context of IoT adoption in health care and enables an evidence-based comparison of theoretical frameworks based on empirical support.

We then visually mapped the structure of each adoption model, such as the TAM and the HBM, using conceptual diagrams. In these figures, each arrow represents a theoretical path, and its thickness reflects the weight. Thicker lines indicate a weight above 0.700, representing paths supported by a high proportion of studies. The numerical values attached to each path represent the average effect size based on the random-effects meta-analysis, along with the corresponding *P* value. This dual representation enables a clearer comparison between the predictive strength (effect size) and consistency (weight) of each construct within and across models.

## Results

### Descriptive Analysis

Papers on IoT health care adoption show an increasing trend, with 89 of the 109 (81.7%) studies in our analysis published between 2020 and 2025, and the earliest published in 2011. According to the SCImago Journal & Country Rank, most papers appeared in Q1 journals (n=63, 57.8%), followed by Q2 (n=36, 33%) and Q3 (n=10, 9.2%), with no papers published in Q4. These studies span major research areas related to health and medicine, information systems, and computer science. In total, 75 unique journals were represented, with *PLoS One* (n=6), *Frontiers in Public Health* (n=5), *Technological Forecasting and Social Change* (n=5), and *International Journal of Environmental Research and Public Health* (n=4) being the most frequently appearing journals (see Table S3 in Multimedia Appendix 1).

**Figure 1:**
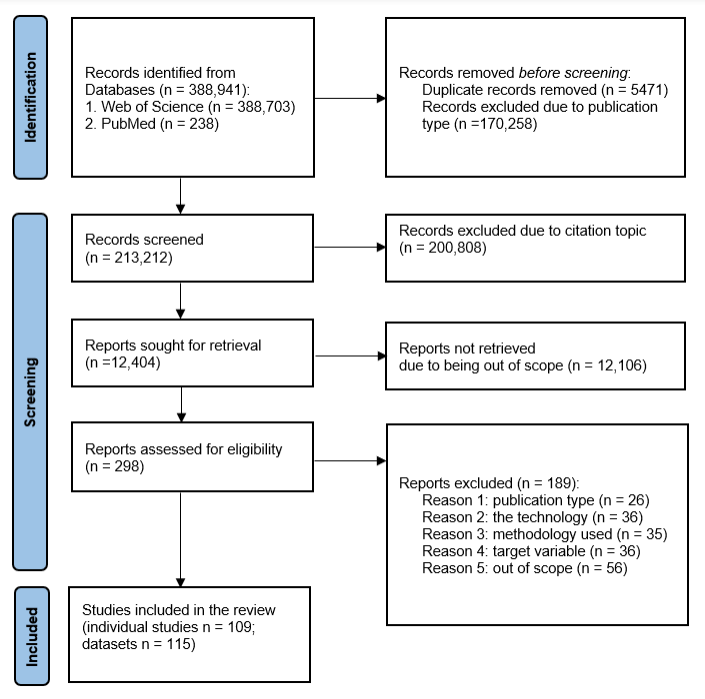
PRISMA (Preferred Reporting Items for Systematic Reviews and Meta-Analyses) flowchart.

In our analysis of 109 studies (see Figure 1), we identified 115 unique datasets totaling 46,508 individuals (see Table S1 in the Multimedia Appendix 1). Studies conducted in China, South Korea, and the United States accounted for a large portion of the total sample and represented the greatest number of publications (see Table 2).

**Table 2:** Number of papers and total sample size per country.

Country	Dataset count (N=115)	Sample size (N=46,508)
China	39	17,068
South Korea	12	6406
The United States	9	5445
India	8	2924
Taiwan	8	1896
The Kingdom of Saudi Arabia	5	1798
Bangladesh	2	1213
Pakistan	3	1194
Turkey	4	1040
Ghana	2	965
Multiple countries	4	1312
Indonesia	1	772
Malaysia	2	628
France	3	515
Iraq	1	465
Oman	2	442
Romania	1	440
The United Arab Emirates	2	431
Switzerland	2	323
Singapore	1	306
Nepal	1	280
Japan	1	233
Italy	1	212
Jordan	1	200

**Figure 2:**
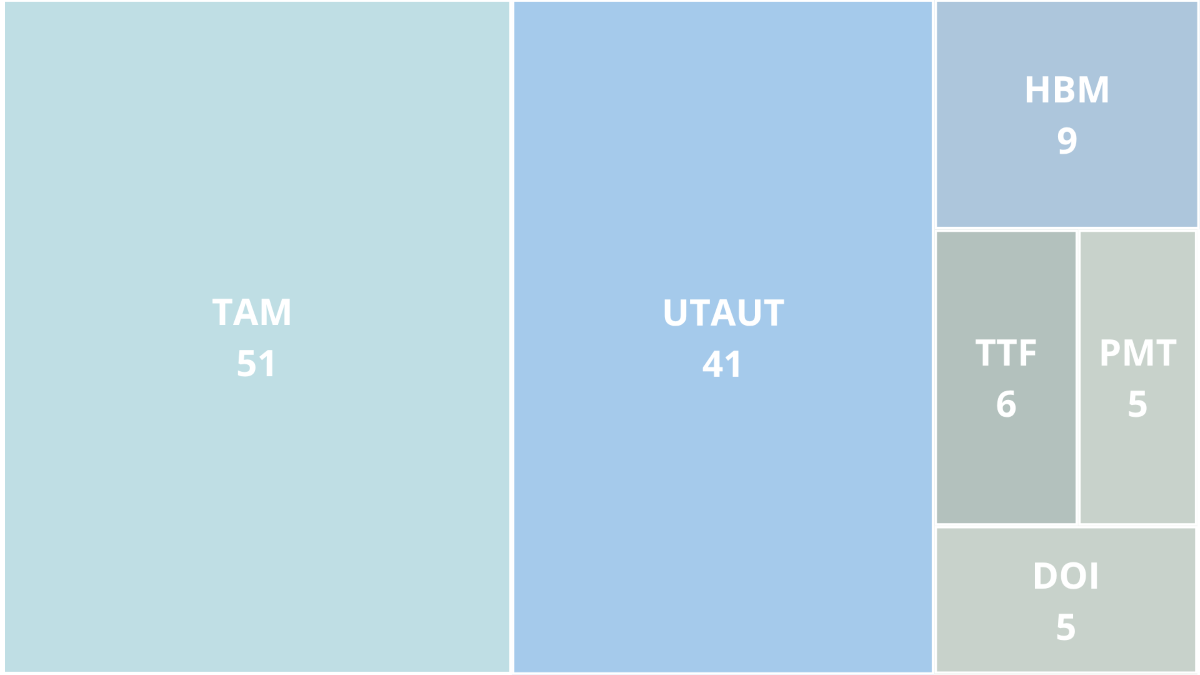
Theoretical foundation of the papers included in our analysis. DOI: Diffusion of Innovation; HBM: Health Belief Model; PCT: Privacy Calculus Theory; PMT: Protection Motivation Theory; TAM: Technology Acceptance Model; TPB: Theory of Planned Behavior; TTF: Task-Technology Fit; UTAUT: Unified Theory of Acceptance and Use of Technology.

Considering the theories addressed in each paper, the TAM and the UTAUT have been extensively examined compared with other theories (see Figure 2). These models serve as the theoretical foundation for 92 of the 109 (84.4%) papers included in our study. Other theories, such as the HBM, PMT, Task-Technology Fit (TTF) Theory, Privacy Calculus Theory, Diffusion of Innovation Theory, and Theory of Planned Behavior, have also been addressed—sometimes as the primary theoretical foundation and other times to extend the TAM or UTAUT.

### Weight Analysis

A weight analysis examines the strength of the relationship between an independent and a dependent variable. The weights of the identified relationships are analyzed and presented in Table 3. The significance of a relationship’s weight is calculated by dividing the number of instances in which the relationship is statistically significant by the total number of studies that investigated it. A weight of 1 indicates that the relationship is significant in all examined studies, whereas a weight of 0 indicates that it is not significant in any of the studies.

**Table 3:** Identified paths with the nonsignificant paths, the significant relationships, the total paths, and the respective weights.

Dependent and independent variables			Significant		Nonsignificant		Total		Weight=significant/total
**Attitude**									
	Effort expectancy	19		4		23		0.826	
	Barriers	4		2		6		0.667	
	Benefits	3		0		3		1	
	Facilitating conditions	4		0		4		1	
	Performance expectancy	20		2		22		0.909	
	Privacy and security	2		1		3		0.667	
	Social influence	6		1		7		0.857	
**Behavioral intention**									
	Aesthetic appeal	4		0		4		1	
	Attitude	27		1		28		0.964	
	Barriers	7		6		13		0.538	
	Benefits	6		0		6		1	
	Compatibility	4		3		7		0.571	
	Effort expectancy	36		23		59		0.61	
	Ethics	2		1		3		0.667	
	Facilitating conditions	20		8		28		0.714	
	Financial cost	12		9		21		0.571	
	Functional congruence	4		1		5		0.8	
	Habit	7		0		7		1	
	Health	3		1		4		0.75	
	Health consciousness	7		4		11		0.636	
	Hedonic motivation	10		5		15		0.667	
	Image	4		2		6		0.667	
	Innovativeness	5		3		8		0.625	
	Perceived severity	2		3		5		0.4	
	Perceived vulnerability	3		5		8		0.375	
	Performance expectancy	68		10		78		0.872	
	Privacy and security	13		13		26		0.5	
	Reliability	5		0		5		1	
	Self-efficacy	10		1		11		0.909	
	Social influence	31		10		41		0.756	
	Technology anxiety	2		4		6		0.333	
	Trust	9		3		12		0.75	
**Actual behavior**									
	Behavioral intention	16		1		17		0.941	
	Effort expectancy	2		1		3		0.667	
	Facilitating conditions	3		0		3		1	
	Health consciousness	2		2		4		0.5	
	Innovativeness	2		1		3		0.667	
	Perceived vulnerability	2		1		3		0.667	
	Performance expectancy	3		1		4		0.75	
	Social influence	3		0		3		1	
**Performance expectancy**									
	Barriers	2		3		5		0.4	
	Compatibility	6		2		8		0.75	
	Convenience	3		0		3		1	
	Effort expectancy	27		4		31		0.871	
	Facilitating conditions	2		2		4		0.5	
	Health consciousness	6		2		8		0.75	
	Image	3		4		7		0.429	
	Innovativeness	4		0		4		1	
	Privacy and security	4		2		6		0.667	
	Reliability	8		3		11		0.727	
	Self-efficacy	7		0		7		1	
	Social influence	8		3		11		0.727	
	Trialability	2		1		3		0.667	
	Trust	3		2		5		0.6	
	Task-technology fit	5		0		5		1	
**Effort expectancy**									
	Compatibility	7		0		7		1	
	Facilitating conditions	6		0		6		1	
	Image	2		2		4		0.5	
	Innovativeness	7		0		7		1	
	Privacy and security	2		2		4		0.5	
	Reliability	4		0		4		1	
	Self-efficacy	7		0		7		1	
	Social influence	3		1		4		0.75	
	Trialability	2		1		3		0.667	
	Task-technology fit	3		0		3		1	
**Task-technology fit**									
	Task characteristics	3		1		4		0.75	
	Technology characteristics	4		0		4		1	

In the context of technology adoption at the individual level, independent variables are considered “well-utilized” if they have been tested at least 5 times. Variables tested fewer than 5 times but with a weight of 1 are regarded as “promising” predictors [31]. To be classified as a “best” predictor, an independent variable must have a weight of 0.800 or higher and must have been examined at least 5 times [31].

In our research, we analyzed the impact of several independent variables on the dependent variables attitude, behavioral intention, actual use, performance expectancy, effort expectancy, and TTF. For the weight analysis, we included relationships that were examined 3 or more times, resulting in 67 relationships and 31 unique predictors that met this criterion. The most studied target variable was behavioral intention, with 25 predictors.

In our research, the relationships considered the “best” predictors for attitude are effort expectancy, performance expectancy, and social influence, as each has more than 5 identified relationships and a weight greater than 0.800. For behavioral intention, the best predictors are attitude, performance expectancy, habit, self-efficacy, functional congruence, reliability, and benefits. Aesthetic appeal, with a perfect weight of 1, is considered a promising predictor of intention due to the limited number of studies. Social influence, facilitating conditions, and trust, although not classified as the best predictors, remain important because their weights exceed 0.700 and are supported by a substantial number of studies. It is also noteworthy that privacy and security, barriers, vulnerability, severity, compatibility, and financial cost yielded more inconsistent results, with many studies reporting statistically nonsignificant findings.

For actual behavior, behavioral intention is the best predictor, while facilitating conditions and social influence are considered promising predictors due to the limited number of studies and their perfect weight of 1. For the target variable performance expectancy, effort expectancy, TTF, and self-efficacy are the best predictors, and convenience and innovativeness are promising predictors. Health consciousness, social influence, reliability, and compatibility, although not classified as the best predictors, remain important because their weights exceed 0.700 and they are supported by multiple studies. For effort expectancy, facilitating conditions, innovativeness, self-efficacy, and compatibility are the best predictors, while reliability and TTF are promising predictors. For the target variable TTF, technology characteristics is identified as a promising predictor.

### Meta-Analysis

The results of the meta-analysis are presented in Table 4 and include all studies that reported standardized path coefficients or *β* values. All the best predictors identified in our study are statistically significant (*P*<.001), except for reliability (*P*=.49) as a predictor of intention, as well as some of the important and promising predictors. Notably, barriers (*P*=.46) is not a significant predictor of attitude. Health, technology anxiety (*P*=.78), financial cost (*P*=.16), and barriers (*P*=.84) are not significant predictors of behavioral intention. For actual behavior, social influence (*P*=.15), innovativeness (*P*=.28), health consciousness (*P*=.61), vulnerability (*P*=.31), and effort expectancy (*P*=.09) are not significant predictors. Privacy and security (*P*=.05) and barriers (*P*=.21) are not significant predictors of performance expectancy, while privacy and security (*P*=.29) and image (*P*=.06) are not significant predictors of effort expectancy. Finally, task characteristics (*P*=.12) is not a significant predictor of TTF.

**Table 4:** Meta-analysis results calculated using a random-effects model and presented back-transformed.

Dependent and independent variables		r/ES^a^	95% CI	*Q* statistic	*z* score	*P* value	*I*^2^ statistic (%)
**Attitude**							
	Barriers	0.069	–0.113 to 0.251	107.606	0.743	.46	95.353
	Benefits	0.466	0.239 to 0.645	224.814	3.789	<.001	99.11
	Effort expectancy	0.286	0.23 to 0.342	153.69	9.549	<.001	86.987
	Facilitating conditions	0.527	0.344 to 0.671	874.478	5.067	<.001	99.657
	Performance expectancy	0.532	0.414 to 0.633	987.838	7.596	<.001	98.077
	Privacy and security	–0.355	–0.618 to –0.093	354.157	–2.653	.008	99.435
	Social influence	0.342	0.182 to 0.483	305.084	4.069	<.001	98.033
**Behavioral intention**							
	Health	0.082	–0.049 to 0.21	18.448	1.233	.22	83.738
	Aesthetic appeal	0.319	0.266 to 0.371	1.314	11.027	<.001	0
	Attitude	0.573	0.454 to 0.672	1855.792	7.853	<.001	98.653
	Barriers	–0.016	–0.171 to 0.139	469.927	–0.2	.84	97.446
	Benefits	0.309	0.092 to 0.497	688.109	2.757	.006	99.273
	Compatibility	0.123	0.081 to 0.165	109.339	5.729	<.001	96.342
	Effort expectancy	0.185	0.134 to 0.235	887.804	7.043	<.001	93.58
	Ethics	0.303	–0.232 to 0.698	143.458	1.117	.26	98.606
	Facilitating conditions	0.198	0.138 to 0.257	274.658	6.318	<.001	90.17
	Financial cost	–0.08	–0.191 to 0.031	541.286	–1.414	.16	96.675
	Functional congruence	0.212	0.165 to 0.259	39.56	8.509	<.001	89.889
	Habit	0.377	0.307 to 0.444	495.521	9.72	<.001	98.789
	Health consciousness	0.298	0.222 to 0.371	4455.165	7.332	<.001	99.798
	Hedonic motivation	0.202	0.174 to 0.23	135.59	13.785	<.001	89.675
	Image	0.201	–0.215 to 0.556	62.391	0.947	.34	93.589
	Innovativeness	0.223	0.147 to 0.297	154.334	5.642	<.001	96.112
	Performance expectancy	0.339	0.295 to 0.381	1212.594	14.281	<.001	93.732
	Privacy and security	–0.11	–0.202 to –0.018	361.354	–2.348	.02	93.912
	Reliability	0.148	–0.266 to 0.516	398.688	0.694	.49	98.997
	Self-efficacy	0.318	0.257 to 0.377	818.281	9.644	<.001	98.9
	Severity	0.12	0.005 to 0.231	8.992	2.04	.04	55.518
	Social influence	0.254	0.189 to 0.317	684.855	7.381	<.001	94.305
	Technology anxiety	–0.013	–0.1 to 0.075	19.306	–0.279	.78	74.102
	Trust	0.294	0.177 to 0.403	666.839	4.769	<.001	98.35
	Vulnerability	0.101	0.009 to 0.191	19.657	2.153	.03	64.389
**Actual behavior**							
	Behavioral intention	0.563	0.427 to 0.674	1139.269	6.912	<.001	98.508
	Effort expectancy	0.353	–0.061 to 0.663	99.717	1.683	.09	97.994
	Facilitating conditions	0.863	0.706 to 0.939	10353.056	5.987	<.001	99.981
	Health consciousness	0.096	–0.267 to 0.436	1594.656	0.511	.61	99.812
	Innovativeness	0.232	–0.188 to 0.581	420.955	1.086	.28	99.525
	Performance expectancy	0.406	0.001 to 0.696	129.825	1.963	.05	98.459
	Social influence	0.31	–0.113 to 0.638	35.827	1.447	.15	94.418
	Vulnerability	0.216	–0.204 to 0.569	615.529	1.008	.31	99.675
**Performance expectancy**							
	Effort expectancy	0.38	0.302 to 0.454	587.488	8.873	<.001	95.064
	Barriers	–0.137	–0.353 to 0.078	396.849	–1.252	.21	98.992
	Compatibility	0.222	0.041 to 0.388	85.162	2.403	.02	92.955
	Facilitating conditions	0.328	0.1 to 0.523	239.597	2.784	.005	98.748
	Health consciousness	0.188	0.021 to 0.345	140.742	2.206	.03	95.026
	Image	0.194	0.001 to 0.373	96.096	1.969	.049	94.797
	Innovativeness	0.279	0.049 to 0.481	115.684	2.364	.02	97.407
	Privacy and security	–0.193	–0.387 to 0.001	919.956	–1.945	.05	99.456
	Reliability	0.309	0.171 to 0.435	82.88	4.258	<.001	87.934
	Self-efficacy	0.578	0.431 to 0.695	531.613	6.52	<.001	99.059
	Social influence	0.315	0.177 to 0.44	121.563	4.349	<.001	91.774
	Trust	0.254	0.046 to 0.441	92.595	2.382	.02	95.68
	Task-technology fit	0.678	0.544 to 0.778	278.406	7.539	<.001	98.563
**Effort expectancy**							
	Compatibility	0.318	0.208 to 0.42	35.374	5.441	<.001	85.865
	Facilitating conditions	0.436	0.343 to 0.521	24.225	8.321	<.001	79.36
	Image	0.147	–0.005 to 0.294	75.386	1.893	.06	97.347
	Innovativeness	0.376	0.287 to 0.458	133.654	7.786	<.001	95.511
	Privacy and security	–0.074	–0.21 to 0.063	329.763	–1.06	.29	99.09
	Reliability	0.46	0.339 to 0.566	45.702	6.747	<.001	93.436
	Self-efficacy	0.604	0.529 to 0.67	1371.419	12.34	<.001	99.635
	Social influence	0.235	0.097 to 0.364	26.199	3.31	<.001	88.549
	Task-technology fit	0.883	0.843 to 0.914	910.714	17.155	<.001	99.78
**Task-technology fit**							
	Task characteristics	0.249	–0.07 to 0.522	460.432	1.537	.12	99.348
	Technology characteristics	0.654	0.429 to 0.803	123.033	4.729	<.001	97.562

^a^r/ES: combined effect size (back-transformed from Fisher *z*).

### Combining Weight and Meta-Analysis Results: The Most Adopted Models in Research

**Figure 3:**
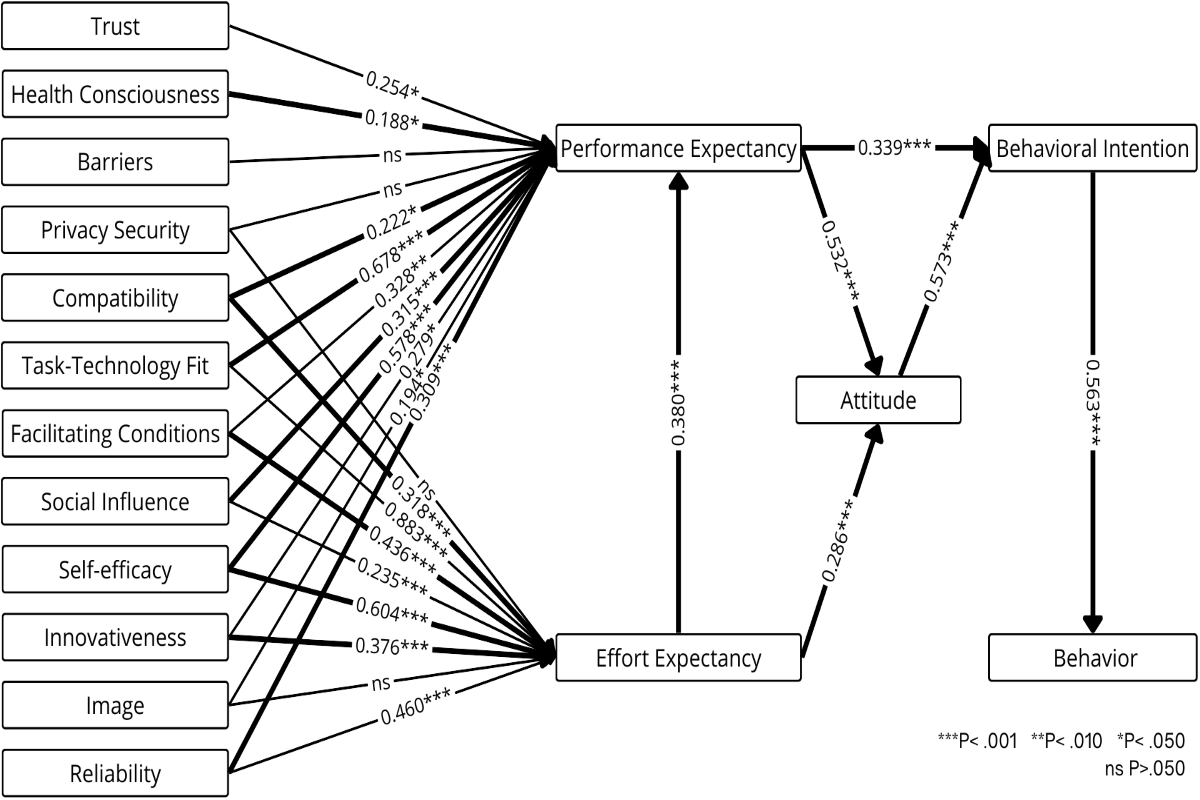
Weight and meta-analysis for the Technology Acceptance Model. Thicker paths indicate relationships with greater weight—that is, the strongest predictors (weight≥0.700). Higher weights are therefore represented by thicker lines. The numbers on the paths denote the mean β coefficients along with their significance levels.

Figure 3 presents the weight and meta-analysis for the TAM, which explains how users adopt and use technology, emphasizing the influence of external variables on perceived usefulness (performance expectancy) and perceived ease of use (effort expectancy), which in turn affect attitudes, behavioral intentions, and actual technology usage [44,45]. Performance expectancy is the best and statistically significant predictor of both attitude (*β*=.532, *P*<.001) and behavioral intention (*β*=.339, *P*<.001). Attitude is the best predictor and has a significant impact on behavioral intention (*β*=.573, *P*<.001), while behavioral intention is the best and significant predictor of actual behavior (*β*=.563, *P*<.001). Effort expectancy is the best predictor and strongly influences performance expectancy (*β*=.380, *P*<.001) and attitude (*β*=.286, *P*<.001). Health consciousness (*β*=.188, *P*=.03), self-efficacy (*β*=.578, *P*<.001), innovativeness (*β*=.279, *P*=.02), and compatibility (*β*=.222, *P*=.02) are significant predictors of performance expectancy, each with a weight above 0.700. Innovativeness (*β*=.376, *P*<.001) and facilitating conditions (*β*=.436, *P*<.001) are significant predictors of effort expectancy, also with weights above 0.700.

**Figure 4:**
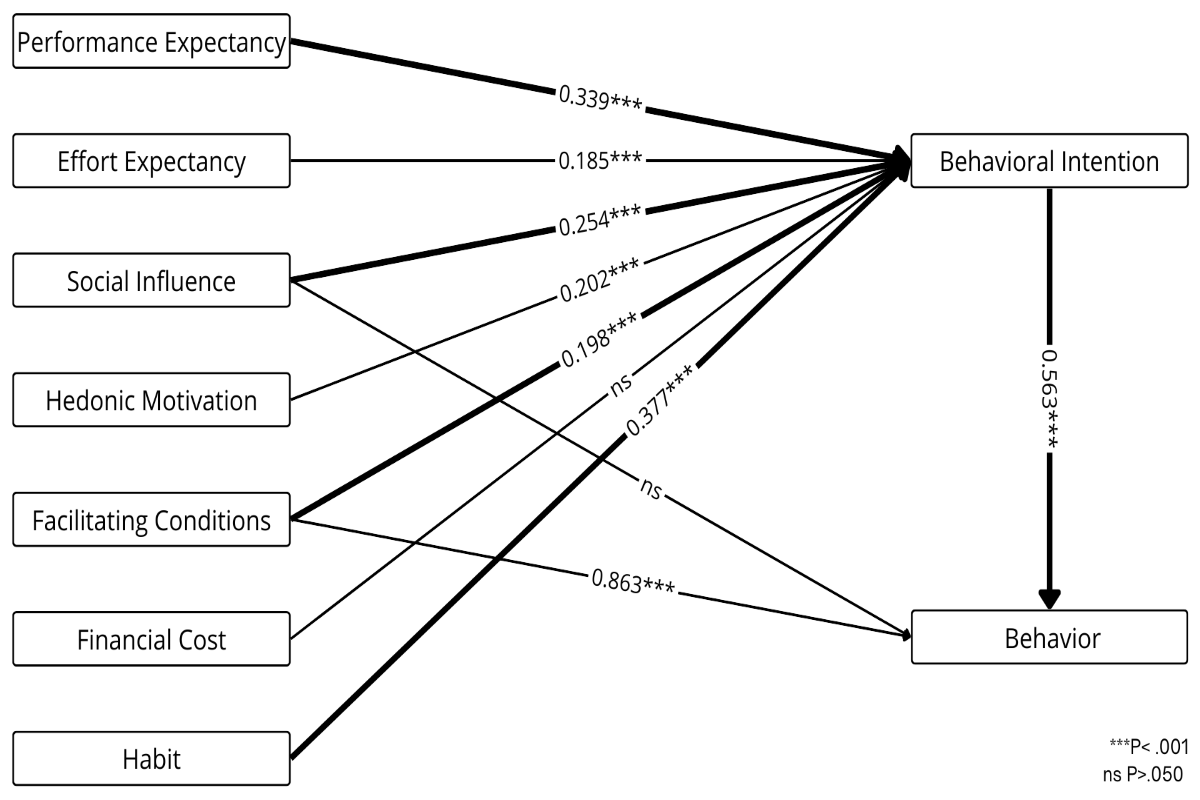
Weight and meta-analysis for the Unified Theory of Acceptance and Use of Technology. Thicker paths indicate relationships with greater weight—that is, the strongest predictors (weight≥0.700). Accordingly, higher weights are represented by thicker lines. The numbers on the paths denote the mean β coefficients along with their significance levels.

Figure 4 presents the weight and meta-analysis for the Unified Theory of Acceptance and Use of Technology (UTAUT), which explains how users adopt and use technology by assessing the impact of key predictors on behavioral intention and actual behavior [46,47]. Facilitating conditions (*β*=.863, *P*<.001) and behavioral intention (*β*=.563, *P*<.001) are significant predictors of actual behavior, while social influence is not. Behavioral intention is significantly influenced by performance expectancy (*β*=.339, *P*<.001), social influence (*β*=.254, *P*<.001), facilitating conditions (*β*=.198, *P*<.001), and habit (*β*=.377, *P*<.001), all with weights above 0.700. Effort expectancy (*β*=.185, *P*<.001) and hedonic motivation (*β*=.202, *P*<.001) are also statistically significant predictors of intention; however, financial cost is not.

**Figure 5:**
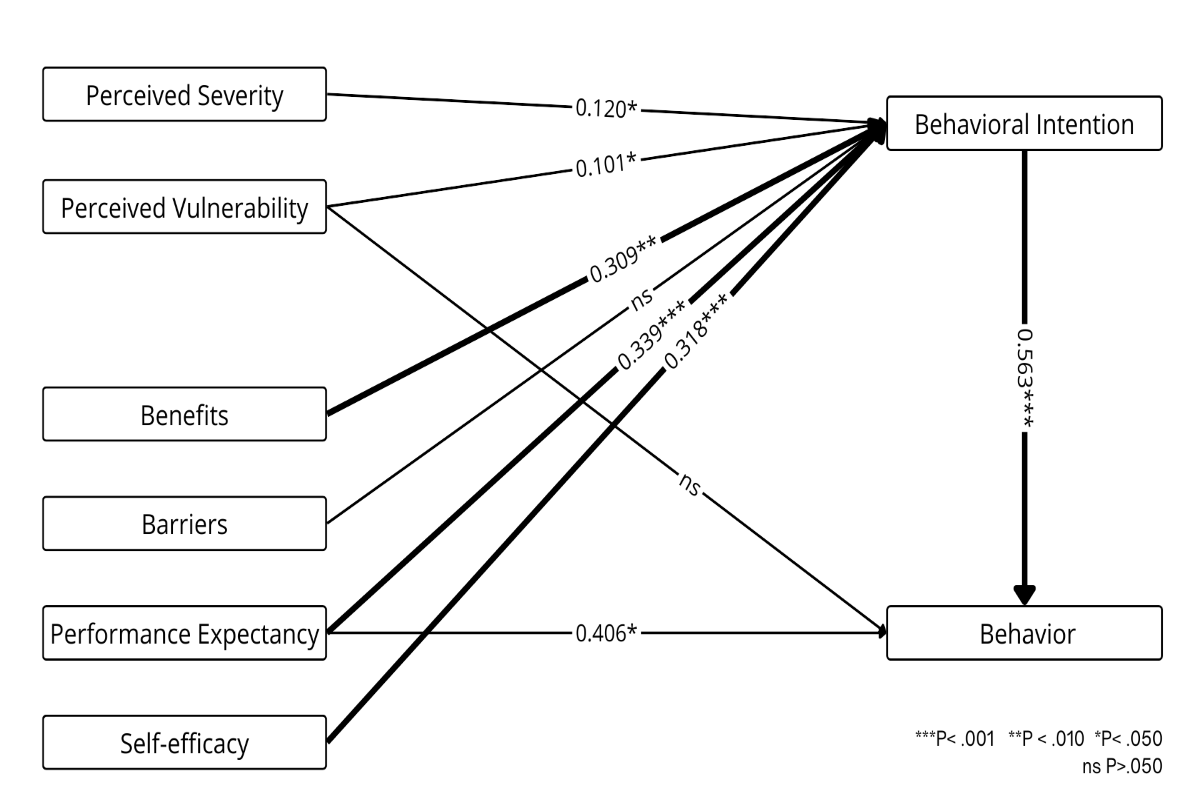
Weight and meta-analysis for the Health Belief Model and Protection Motivation Theory. Thicker paths indicate relationships with greater weight—that is, the strongest predictors (weight≥0.700). Accordingly, higher weights are represented by thicker lines. The numbers on the paths denote the mean β coefficients along with their significance levels.

Figure 5 presents the weight and meta-analysis combining the HBM and PMT, which explain individuals’ engagement in health-related behaviors. Both models emphasize the role of perceived threat, such as vulnerability and severity, and the evaluation of coping strategies, such as benefits, barriers, response efficacy, and self-efficacy, in shaping motivation to take protective or preventive actions [48-50]. The results indicate that, compared with other technology adoption models, the predictive power of health-related constructs is weaker and less consistent. Severity (*β*=.120, *P*=.04) and vulnerability (*β*=.101, *P*=.03) have weak weights on behavioral intention and exert a small but significant impact. Performance expectancy (*β*=.339, *P*<.001) and self-efficacy (*β*=.318, *P*<.001), which are also used in other technology-related adoption models, are the best predictors and have a significant impact on behavioral intention. Barriers do not have a significant impact (*P*=.84), whereas benefits exhibit a strong, statistically significant effect (*β*=.309, *P*=.006). It is also relevant to mention 2 additional predictors directly related to the health context but not part of the key components of these theories. First, health condition, which refers to the perception of having good health, has a weak and nonsignificant impact (*P*=.22) on intention. Second, health consciousness has a statistically significant impact on intention (*β*=.298, *P*<.001) but does not significantly influence behavior (*P*=.61).

**Figure 6:**
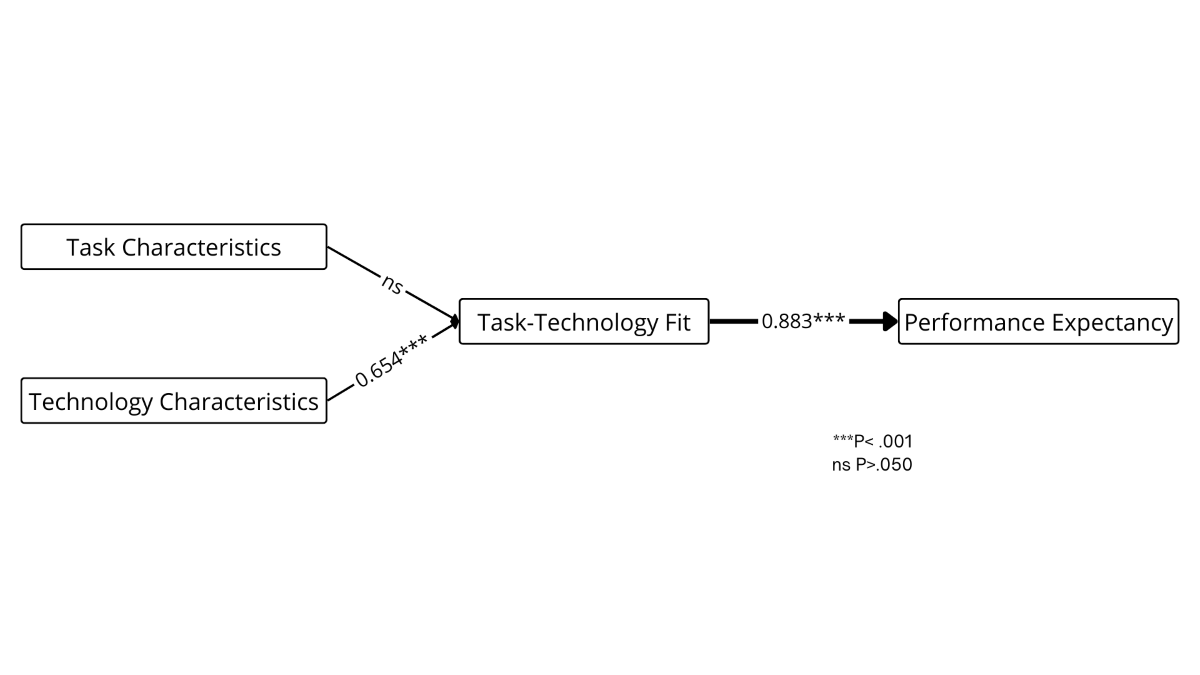
Weight and meta-analysis for the Task–Technology Fit model. Thicker paths indicate relationships with greater weight—that is, the strongest predictors (weight≥0.700). Accordingly, higher weights are represented by thicker lines. The numbers on the paths denote the mean β coefficients along with their significance levels. ns: not significant.

Figure 6 illustrates the TTF model, which examines how well technology aligns with users’ tasks to enhance perceived usefulness and adoption [51]. Only part of the theory is presented, as it remains understudied in the context of IoT in health care. The results show that TTF is a significant predictor of performance expectancy (*β*=.883, *P*<.001) while being classified as a promising predictor. The studies suggest that task characteristics do not have a significant impact on the fit between the task and the technology. However, technology characteristics are a promising and significant predictor (*β*=.554, *P*<.001).

**Figure 7:**
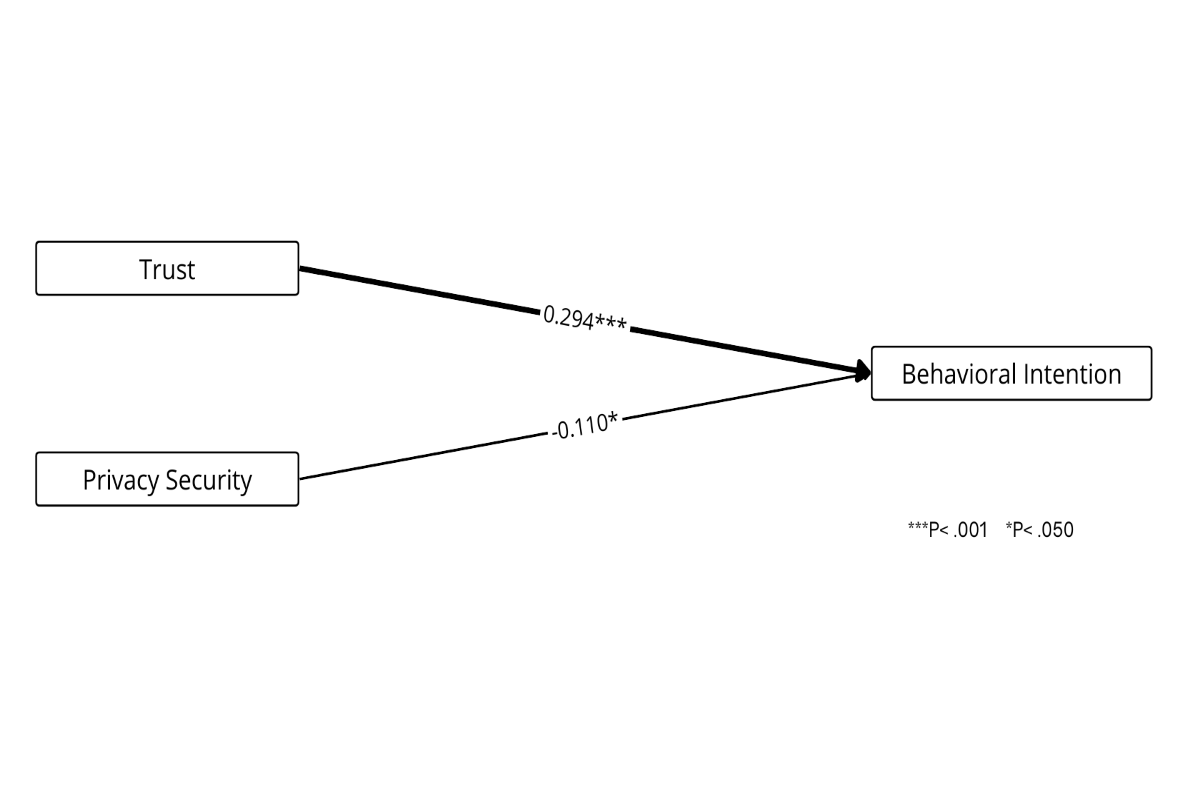
Weight and meta-analysis for Privacy Calculus Theory. Thicker paths indicate relationships with greater weight—that is, the strongest predictors (weight≥0.700). Accordingly, higher weights are represented by thicker lines. The numbers on the paths denote the mean β coefficients along with their significance levels.

Figure 7 presents the weight and meta-analysis of the Privacy Calculus Theory, which explores the trade-off between benefits and privacy [52]. The results indicate that trust (*β*=.294, *P*<.001) has a significant positive influence on behavioral intention and is classified as the best predictor. Privacy and security (*β*=–.110, *P*=.02) exhibits a significant negative effect on behavioral intention; however, the weight is small, suggesting that privacy and security concerns may not be a strong inhibitor of adoption.

**Figure 8:**
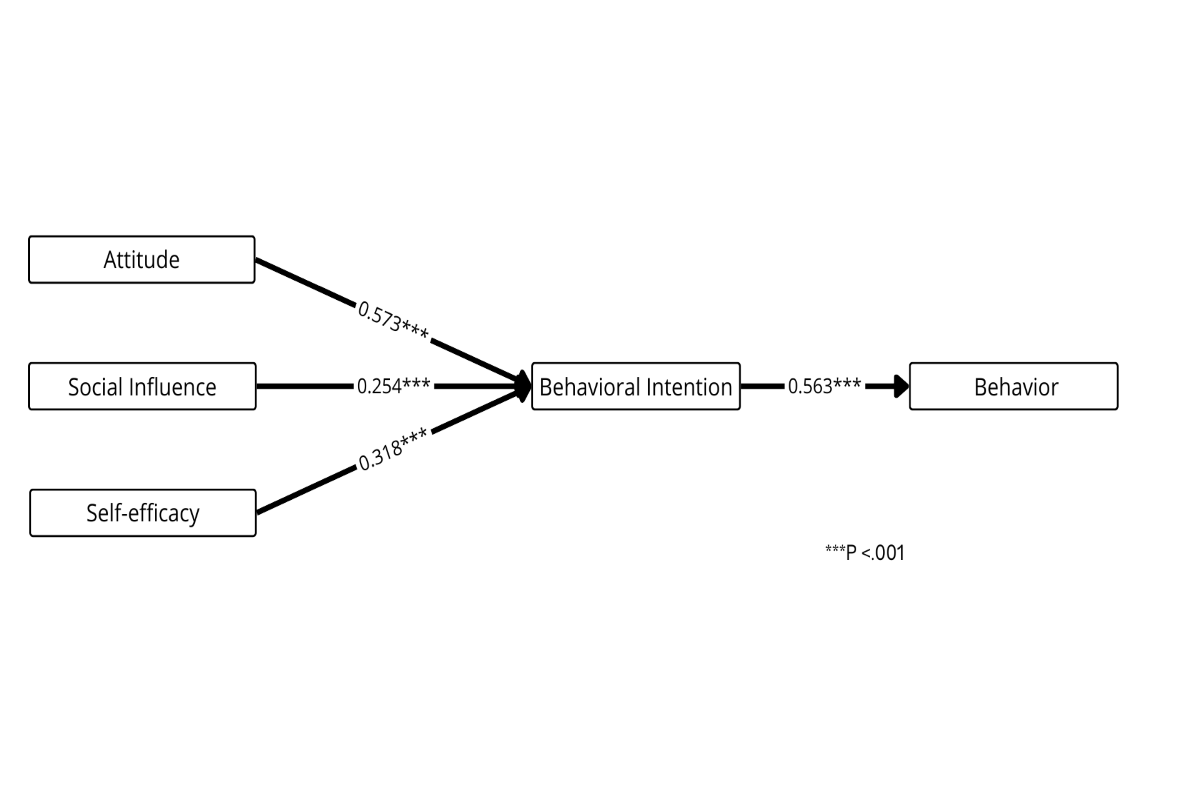
Weight and meta-analysis for the Theory of Planned Behavior. Thicker paths indicate relationships with greater weight—that is, the strongest predictors (weight≥0.700). Accordingly, higher weights are represented by thicker lines. The numbers on the paths denote the mean β coefficients along with their significance levels.

Figure 8 presents the weight and meta-analysis of the Theory of Planned Behavior, which posits that attitude, subjective norms, and behavioral control influence behavioral intention and, subsequently, behavior [53]. Both attitude (*β*=.573, *P*<.001) and self-efficacy (*β*=.318, *P*<.001) are the best and strongest predictors of behavioral intention.

**Figure 9:**
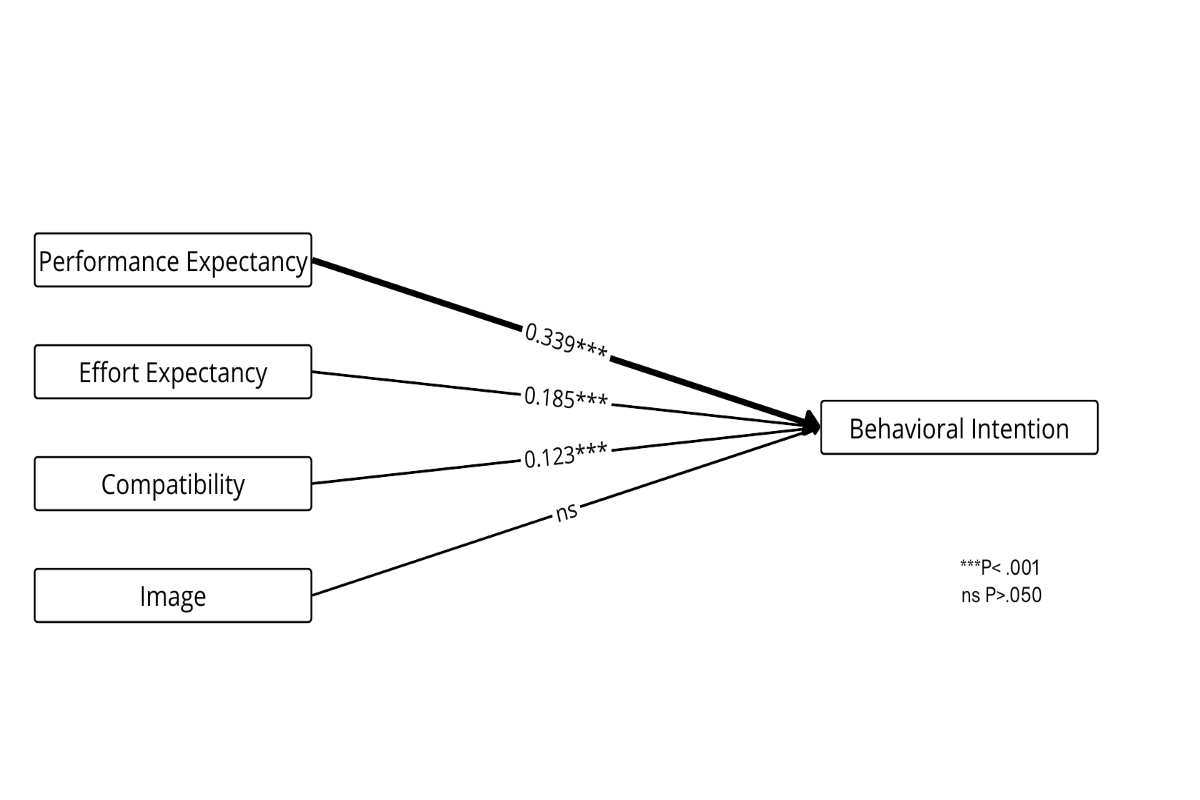
Weight and meta-analysis for the Diffusion of Innovation theory. Thicker paths indicate relationships with greater weight—that is, the strongest predictors (weight≥0.700). Accordingly, higher weights are represented by thicker lines. The numbers on the paths denote the mean β coefficients along with their significance levels.

Figure 9 presents the weight and meta-analysis of the Innovation Diffusion Theory, which explains the diffusion of new technologies through 5 dimensions [54]. Relative advantage (performance expectancy; *β*=.339, *P*<.001), complexity (effort expectancy; *β*=.185, *P*<.001), and compatibility (*β*=.123, *P*<.001) were found to be significant predictors. Image was not a significant predictor, and trialability and observability have not been sufficiently studied in the literature.

### Evaluation of Publication Bias

This section evaluates the presence of publication bias and assesses the normality of the datasets used in the meta-analysis to ensure the reliability of the synthesized findings. Publication bias refers to the tendency for studies with significant or positive results to be more likely to be published, potentially skewing meta-analytic outcomes [55]. To ensure the robustness of our findings, we evaluated publication bias following the approach of Harrison et al [55], which suggests that a single criterion can provide a more sensitive and appropriate test. We focused our analysis on one of the most widely examined relationships in our dataset: the relationship between performance expectancy and behavioral intention, which was reported in 77 studies (Table 5).

**Table 5:** Studies (n=77) showing the effect size (z), SE (z), sample size, z score, Q component, significance of the paths between performance expectancy and behavioral intention, and the country.

Subgroup and effect size (*Z*)			SE (*Z*)		Sample size	*z* score		*Q* component		Significance		Country
**Group 1**												
	0.268	0.051		387		5.965	2.350		Significant		China	
	0.604	0.050		397		13.455	26.161		Significant		China	
	0.387	0.080		158		7.933	0.258		Significant		China	
	0.165	0.046		469		3.718	15.264		Significant		China	
	0.086	0.053		357		1.908	23.979		Significant		China	
	0.230	0.051		386		5.113	5.199		Significant		China	
	0.727	0.065		243		15.616	34.684		Significant		China	
	0.224	0.036		769		5.122	11.555		Significant		China	
	0.186	0.058		304		4.074	7.740		Significant		China	
	0.149	0.113		81		2.702	3.039		Nonsignificant		China	
	0.333	0.071		201		7.018	0.037		Significant		China	
	0.472	0.050		406		10.530	6.373		Significant		China	
	0.205	0.056		325		4.504	6.462		Significant		China	
	0.198	0.054		345		4.362	7.588		Significant		China	
	0.217	0.086		139		4.372	2.267		Significant		China	
	0.950	0.084		146		19.257	52.169		Significant		China	
	0.406	0.065		237		8.704	0.827		Significant		China	
	0.471	0.072		197		9.914	3.014		Significant		China	
	0.519	0.065		239		11.139	7.032		Significant		China	
	0.180	0.047		462		4.039	12.734		Significant		China	
	0.105	0.071		201		2.223	11.509		Nonsignificant		China	
	0.289	0.040		624		6.567	2.068		Significant		China	
	0.286	0.064		247		6.145	0.907		Significant		China	
	0.377	0.039		668		8.591	0.615		Significant		China	
	0.446	0.051		386		9.927	3.830		Significant		China	
	0.157	0.043		552		3.560	19.651		Nonsignificant		China	
	0.258	0.047		450		5.774	3.535		Significant		China	
	0.401	0.096		111		7.772	0.324		Significant		China	
	1.040	0.077		171		21.531	80.869		Significant		China	
	0.586	0.028		1292		13.583	73.940		Significant		China	
	0.224	0.046		475		5.028	7.120		Significant		China	
	0.236	0.037		725		5.401	8.764		Significant		China	
**Group 2**												
	0.422	0.033		913		9.720	5.253		Significant		Bangladesh	
	0.586	0.075		181		12.212	10.210		Significant		France	
	0.132	0.062		267		2.855	12.172		Nonsignificant		France	
	0.202	0.125		67		3.478	1.342		Nonsignificant		France	
	0.633	0.070		206		13.382	16.646		Significant		Germany, the United States, the United Kingdom, and Canada	
	0.102	0.056		320		2.249	18.892		Nonsignificant		Ghana	
	0.319	0.039		645		7.273	0.469		Significant		Ghana	
	0.401	0.050		400		8.939	1.190		Significant		India	
	0.421	0.086		139		8.473	0.761		Significant		India	
	0.283	0.043		534		6.404	2.116		Significant		India	
	0.150	0.052		372		3.330	14.228		Significant		India	
	0.119	0.082		153		2.418	7.793		Nonsignificant		India	
	0.306	0.065		238		6.569	0.381		Significant		India	
	0.415	0.036		772		9.512	3.647		Significant		Indonesia	
	0.549	0.054		341		12.120	13.905		Significant		Iraq	
	0.245	0.069		212		5.191	2.162		Significant		Italy	
	0.413	0.066		233		8.841	1.017		Significant		Japan	
	0.265	0.071		200		5.587	1.307		Significant		Jordan	
	0.205	0.051		389		4.556	7.746		Significant		Korea	
	0.321	0.080		159		6.572	0.105		Significant		Korea	
	0.084	0.029		1158		1.948	79.454		Nonsignificant		Korea	
	0.239	0.060		280		5.209	3.172		Significant		Nepal	
	0.190	0.063		259		4.112	6.247		Significant		Oman	
	0.321	0.045		495		7.220	0.331		Significant		Pakistan	
	0.284	0.046		486		6.401	1.860		Significant		The Kingdom of Saudi Arabia	
	0.229	0.046		473		5.145	6.496		Significant		The Kingdom of Saudi Arabia	
	0.189	0.063		256		4.085	6.256		Significant		The Kingdom of Saudi Arabia	
	0.553	0.046		477		12.441	20.278		Significant		South Korea	
	0.574	0.045		487		12.910	24.967		Significant		South Korea	
	0.523	0.034		877		12.020	27.229		Significant		South Korea	
	0.518	0.097		110		10.014	3.141		Significant		Switzerland	
	0.485	0.055		335		10.682	6.343		Significant		Taiwan	
	0.393	0.061		268		8.519	0.575		Significant		Taiwan	
	0.149	0.047		458		3.346	17.727		Significant		Taiwan	
	0.321	0.091		125		6.340	0.082		Significant		Taiwan	
	0.245	0.113		81		4.436	0.807		Significant		Taiwan	
	0.688	0.065		243		14.796	28.070		Significant		Turkey	
	0.041	0.049		426		0.917	39.467		Nonsignificant		Turkey	
	0.230	0.099		106		4.416	1.398		Significant		The United Arab Emirates	
	0.141	0.050		407		3.143	17.070		Nonsignificant		The United States	
	1.157	0.052		376		25.681	244.930		Significant		The United States	
	0.167	0.060		277		3.619	8.873		Significant		The United States	
	0.266	0.092		120		5.227	0.756		Significant		The United States	
	0.848	0.047		450		19.012	112.411		Significant		The United States	
	0.321	0.056		322		7.045	0.215		Significant		Worldwide	

**Figure 10:**
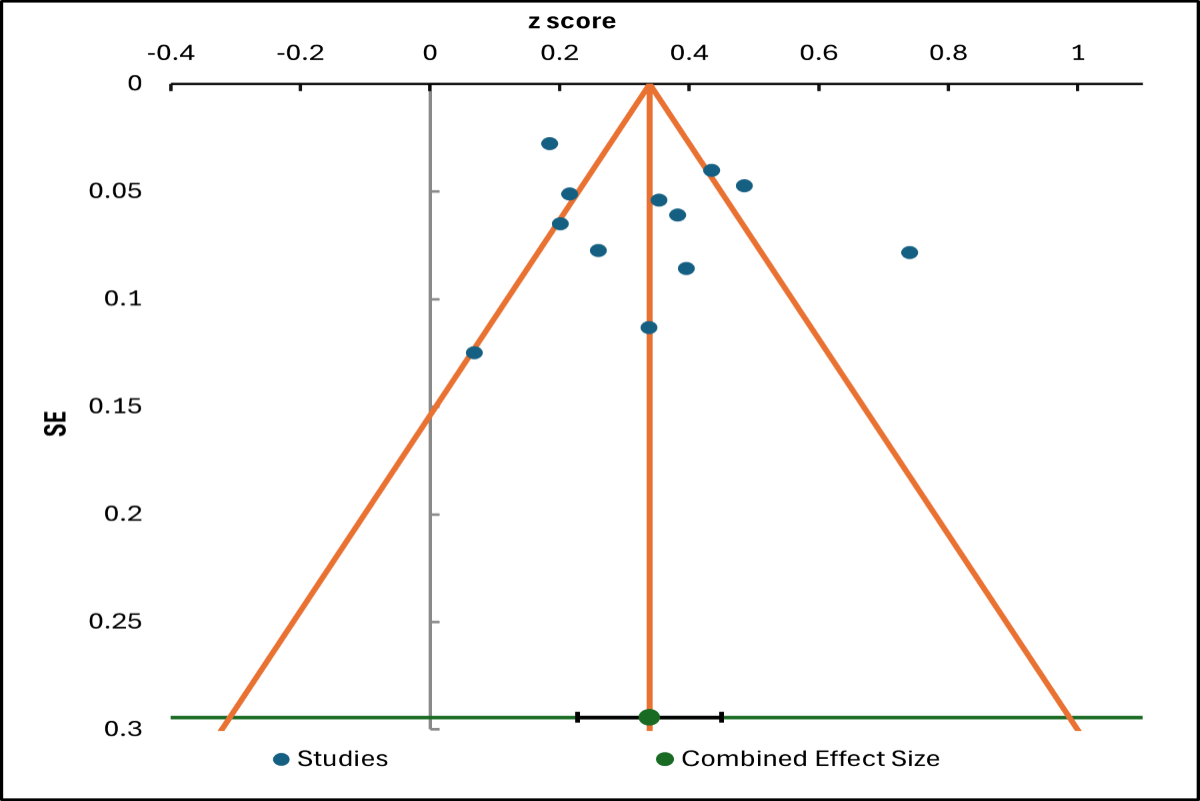
Funnel plot of studies examining the relationship between performance expectancy and behavioral intention.

To assess the presence of small-study effects and potential publication bias, a funnel plot was generated, and an Egger regression test was conducted. The funnel plot was constructed to visually evaluate publication bias [42], with the SE plotted on the y-axis instead of sample size, as this enhances the detection of asymmetry [36]. In an ideal funnel plot, symmetry is expected, with smaller studies exhibiting larger SE scattered evenly on both sides of the pooled effect size. In Figure 10, the studies display a somewhat asymmetrical distribution. Larger studies cluster near the combined effect size at the top of the funnel, while smaller studies show greater dispersion, potentially indicating publication bias or underlying heterogeneity. The trim-and-fill method estimates the number of potentially missing studies—often those with nonsignificant or negative results—and imputes them to generate an adjusted combined effect size. In this case, the imputed effect size is slightly smaller than the original estimate, suggesting that the observed meta-analytic effect may be modestly inflated due to the absence of smaller, less favorable studies.

To statistically assess funnel plot asymmetry, we applied the Egger regression test [42] to evaluate whether smaller studies tend to report larger effect sizes, which can indicate potential publication bias (see Table 6). The test examines the relationship between effect sizes and their SEs to detect small-study effects. The results of the regression analysis showed that the intercept was not statistically significant (*α*=.381, *P*=.81), indicating no evidence of funnel plot asymmetry or publication bias. However, the slope coefficient was statistically significant (*β*=.327, *P*<.001), suggesting a positive association between study precision and effect size. While this does not indicate publication bias, it may reflect genuine heterogeneity among the included studies.

The *I*^2^ statistic, which quantifies the proportion of total variation across studies attributable to true heterogeneity rather than sampling error [41], revealed a very high degree of heterogeneity (*I*^2^>93%). This indicates that most of the variability in effect sizes reflects real differences across studies rather than random sampling error. These differences may arise from variations in study design, measurement tools, participant demographics, cultural contexts, or theoretical frameworks used across the included studies. To further explore the sources of heterogeneity, a subgroup analysis was conducted.

**Table 6:** Egger‐type test for small‐study bias, using Excel’s Data Analysis Regression Tool, which uses standard ordinary least squares regression.

Regression	Coefficients	SE	*t* test (*df*)	*P* value	95% CI
Intercept (*α*)	0.381	1.544	0.247 (75)	.81	–2.695 to 3.457
Slope (*β*)	0.327	0.081	4.044 (75)	<.001	0.166 to 0.489

The subgroup analysis examined whether effect sizes differed between studies conducted in China and those conducted in other countries. By comparing studies from China with those from other regions, we aimed to evaluate potential regional biases, given that a large proportion of the included studies were conducted in China. The results, presented in Table 7, indicate that geographic location has little influence on the overall effect size, as both subgroups exhibit similar results. The combined effect size for studies conducted in China is 0.340 (95% CI 0.272-0.404), while for studies in other countries, it is 0.336 (95% CI 0.279-0.390). However, heterogeneity remained very high in both groups (China: *I*^2^=93%; other countries: *I*^2^=94%), indicating substantial variability even within each subgroup. Therefore, the subgroup analysis addresses concerns about potential bias from the large proportion of studies conducted in China, confirming that the results are largely stable across regions.

**Table 7:** Subgroup comparison between China and the other countries in our sample.

Subgroup	Effect size	*P* value	95% CI	*I*^2^ (%)
China	0.340	<.001	0.272-0.404	92.984
Other countries	0.336	<.001	0.279-0.390	94.355

## Discussion

### Principal Findings

**Figure 11:**
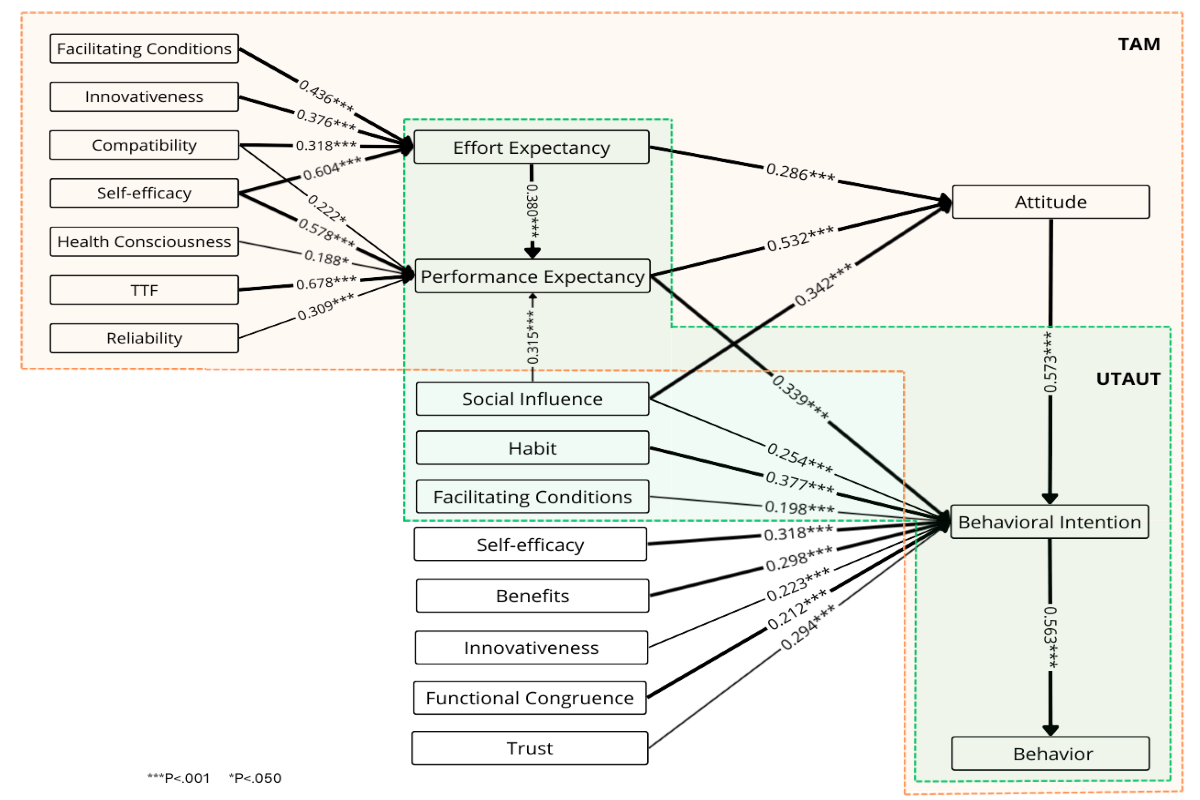
Best predictors identified in the weight analysis, along with their statistically significant mean β coefficients from the meta-analysis. The strength (weight) of each predictor is represented by the thickness of the line connecting the predictor to the target variable, with thicker lines indicating stronger predictors. The green box denotes constructs from the Unified Theory of Acceptance and Use of Technology framework, and the orange box denotes constructs from Technology Acceptance Model.

The study of IoT adoption in health care reveals a diverse landscape of constructs and relationships, providing a comprehensive overview of the factors driving IoT adoption. This study synthesized findings from 109 papers and 115 datasets across various regions, including China, South Korea, the United States, and India, with most studies published in high-ranking journals. The combined weight and meta-analysis identified the best predictors and examined the adoption models most frequently used in IoT health care. Figure 11 highlights the strongest and most consistent predictors, integrating the results of both the meta-analysis and weight analysis.

Technology acceptance models, such as UTAUT and TAM, have been widely and successfully applied in the context of IoT in health care [56,57], which is unsurprising given that these are the most commonly used technology adoption models, highly cited, and successfully applied across diverse fields and contexts [58,59]. Compared with models such as UTAUT and TAM, where predictors such as performance expectancy and facilitating conditions consistently exhibit strong and reliable effects, the HBM and PMT models struggle to establish robust relationships with behavioral intention. This suggests that relying solely on health-related constructs or health behavior models may not be sufficient to explain health care technology adoption. Therefore, other individual factors, such as innovativeness, external factors, such as social influence, and technological factors, such as performance expectancy, may play a more decisive role [60,61]. Integrating context-specific health variables into robust models such as TAM or UTAUT can, however, provide additional insights. For example, individuals with strong health motivation or health consciousness tend to exhibit higher levels of performance expectancy from IoT health care technologies [62,63].

The findings highlight several key factors influencing effort expectancy and performance expectancy, both of which are central to users’ attitudes toward technology. For effort expectancy, the most influential factor was self-efficacy, indicating that individuals who feel more confident in their ability to use the technology tend to perceive it as easier to operate [64,65]. Other important contributors include facilitating conditions, innovativeness, and compatibility, suggesting that a supportive environment, openness to new technologies, and alignment with users’ existing values and practices all help reduce the perceived effort required to use IoT in health care [66,67]. For performance expectancy, TTF emerged as the dominant influence, highlighting that when users perceive a strong alignment between the technology and the tasks they need to perform, they are more likely to view it as useful [68,69]. Additionally, health consciousness, self-efficacy, reliability, and compatibility played significant roles, emphasizing the importance of personal health concerns, confidence in usage, trust in the system’s dependability, and alignment with users’ existing values and practices [63,70]. Together, these findings underscore the relevance of both individual and contextual factors in shaping users’ perceptions of a technology’s usefulness and ease of use.

Regarding individuals’ IoT health care technology adoption journey, the findings reveal that a positive attitude is crucial for successful adoption [71,72]. Efforts to cultivate positive perceptions can be made by leveraging the influence of important figures in individuals’ lives and by emphasizing the ease of use and the potential for improved health care outcomes [73-75]. When individuals hold a positive perception of IoT health care, they are more likely to intend to use it, which in turn positively influences actual usage [76,77]. To further enhance behavioral intention, the effectiveness of IoT health care solutions and the encouragement of health care professionals, family, and friends should be leveraged [78,79]. Additionally, individuals’ willingness to try new technologies plays a significant role, as more innovative users are more likely to adopt IoT solutions [80,81].

Trust plays a decisive role in shaping behavioral intentions, reinforcing the notion that users are willing to trade some level of privacy if they perceive a system as secure and reliable [60]. Previous literature has found that individuals are often reluctant to adopt digital health or IoT technologies when they do not trust the provider [82,83]. This perspective may help contextualize the inconsistent results observed for privacy as a predictor of behavioral intention, as a notable proportion of studies reported nonsignificant relationships. Similarly, predictors such as barriers, vulnerability, and financial cost also exhibited higher frequencies of nonsignificant findings in our analysis. These inconsistencies may reflect how these constructs interact with—or are influenced by—the presence of stronger enabling factors. For instance, high perceived usefulness and trust may diminish the observed effects of barriers such as financial cost and privacy, as these factors may become less salient in users’ perceptions.

### Theoretical Implications

This study makes several contributions to the theoretical understanding of IoT health care adoption by synthesizing findings from diverse quantitative studies and adoption models. The results reinforce the importance of established models such as TAM and UTAUT. They also suggest that integrating variables from other theories—such as health consciousness, innovativeness, and trust—into traditional technology acceptance frameworks can provide deeper insights into how individuals adopt IoT in health care. Behavioral intention is the most studied target variable, while attitude and actual behavior remain underexplored, indicating a gap in existing research on these critical components of the adoption process.

Researchers should further investigate several promising but underexplored predictors that showed perfect weight, suggesting strong yet preliminary evidence of their relevance, to establish their broader applicability. For instance, regarding behavioral intention, the aesthetic appeal of health care technologies shows potential as a strong predictor. For actual behavior, facilitating conditions and social influence are promising predictors that warrant further exploration. In the case of the underexplored TTF theory, technology characteristics appear to be a promising predictor. For performance expectancy, convenience and innovativeness are promising predictors, while for effort expectancy, reliability and TTF show potential as predictors deserving additional investigation.

By contrast, several predictors demonstrated limited or inconsistent relevance to the adoption of IoT in health care. For the outcome attitude, barriers did not have a statistically significant effect. For behavioral intention, predictors such as privacy and security, barriers, vulnerability, severity, compatibility, and financial cost produced inconsistent findings, with many studies reporting nonsignificant results. Specifically, health, technology anxiety, financial cost, and barriers were frequently not significant predictors of behavioral intention. When predicting actual behavior, variables such as social influence, innovativeness, health consciousness, vulnerability, and effort expectancy often failed to reach statistical significance. Regarding performance expectancy, both privacy and security and barriers were not consistently significant, and for effort expectancy, privacy and security and image did not show meaningful effects.

Our findings indicate that regional differences alone do not fully explain the heterogeneity of results. Therefore, when applying the findings of this study, we recommend refining theoretical models to account for contextual factors and implementing practical strategies aligned with the strongest predictors identified, such as performance expectancy and self-efficacy, which can enhance adoption across different settings. Future adoption studies would benefit from incorporating context-specific factors that capture cultural and health care system differences, enabling a better understanding of how these contextual variables influence target outcomes, as either control or moderator variables.

Several regions, particularly in Africa, South America, and Europe, remain underrepresented, highlighting a gap in the literature and the need for future research in diverse settings to improve the generalizability and equity of evidence regarding IoT adoption in health care. Finally, combining qualitative methods, such as interviews and focus groups, is recommended to gain deeper insights. This mixed methods approach can provide a better understanding of user perceptions and experiences, bridging the gap between quantitative results and the complex realities of technology adoption [84,85].

### Practical Implications

The findings of this study provide actionable insights for practitioners, policy makers, and technology developers seeking to enhance IoT health care adoption. Key drivers—such as performance expectancy, self-efficacy, social influence, functional congruence, trust, habit, facilitating conditions, benefits, and innovativeness—consistently shape behavioral intention. For example, developers can focus on creating intuitive designs and user-friendly interfaces while emphasizing tangible performance benefits. Health care providers and policy makers can leverage trusted individuals, such as doctors and family members, to encourage adoption.

The availability of resources and infrastructure that enable and support technology use—such as access to devices, technical support, internet connectivity, and integration with health care systems—plays an important role in adoption, as it reduces barriers for individuals starting to use IoT in health care [69,78]. Furthermore, adhering to robust data protection frameworks that ensure transparency from all entities handling health-related data aligns implementation with national and regional regulatory standards and fosters user trust [83,86]. Finally, targeting innovative individuals who are more likely to adopt IoT health care technologies or who already have the habits and skills to use them can further promote technology adoption.

### Limitations and Future Research

This study has several limitations that warrant consideration. Our findings reveal a high level of heterogeneity, which is not fully explained by regional differences; therefore, the pooled estimates should be interpreted with caution. Future research should investigate additional factors that may account for this heterogeneity, such as study design, population characteristics, or model specification, and conduct moderator analyses to better address variability. Additionally, China accounts for a large proportion of the included studies, while several regions—particularly in Africa, South America, and Europe—remain underrepresented. As such, we caution against overgeneralizing our findings to all global contexts. Additionally, while this study synthesizes quantitative findings, it excludes qualitative research, which could provide deeper insights into contextual variability and user experiences influencing adoption. This exclusion may contribute to inconsistencies in the evidence, particularly for understudied predictors such as privacy concerns, perceived vulnerability, and financial cost, which often showed nonsignificant results. Future research should consider integrative literature reviews that include qualitative studies to better capture the nuanced interplay of individual, cultural, and technological factors.

### Conclusions

Our comprehensive meta- and weight analysis of 115 unique datasets on IoT health care adoption revealed several significant predictors for the adoption of IoT health care technologies. Behavioral intention emerged as the most frequently studied target variable. By contrast, attitude, actual behavior, performance expectancy, effort expectancy, and TTF remain comparatively understudied, with very few paths examined more than 5 times. While adoption theories from the information systems field, such as UTAUT and TAM, are predominantly used, integrating context-specific factors or combining constructs from different theoretical models can provide deeper insights into IoT health care adoption and further support the adoption process.

All the best predictors identified in our study were statistically significant, with the exception of reliability as a predictor of behavioral intention. For the target variable attitude, the strongest predictors were effort expectancy, performance expectancy, and social influence, while barriers did not have a statistically significant effect. Regarding behavioral intention, the most consistent and significant predictors were attitude, performance expectancy, habit, self-efficacy, functional congruence, reliability, and benefits. In addition, social influence, facilitating conditions, and trust demonstrated strong weights above 0.700, while aesthetic appeal was considered a promising predictor due to the limited number of studies. Conversely, variables such as privacy and security, barriers, vulnerability, severity, compatibility, and financial cost showed inconsistent results, with a high incidence of statistically nonsignificant findings. Specifically, health, technology anxiety, financial cost, and barriers were not statistically significant predictors of behavioral intention.

For actual behavior, behavioral intention emerged as the best predictor, while facilitating conditions and social influence were considered promising. However, social influence, innovativeness, health consciousness, vulnerability, and effort expectancy did not reach statistical significance for behavior. Regarding performance expectancy, effort expectancy, TTF, and self-efficacy were the best predictors, followed by health consciousness, social influence, reliability, and compatibility as strong predictors, while convenience and innovativeness appeared as promising. Privacy and security and barriers, however, were not statistically significant predictors of performance expectancy. For effort expectancy, the most consistent predictors were facilitating conditions, innovativeness, self-efficacy, and compatibility, with reliability and TTF considered promising predictors; privacy and security and image did not show significant effects. Lastly, for the target variable TTF, technology characteristics emerged as a promising predictor, whereas task characteristics were not statistically significant.

## Appendix

Multimedia Appendix 1 Search strategy.

## Abbreviations

HBM: Health Belief Model
IoT: Internet of Things
PMT: Protection Motivation Theory
PRISMA: Preferred Reporting Items for Systematic Reviews and Meta-Analyses
TAM: Technology Acceptance Model
TTF: task-technology fit
UTAUT: Unified Theory of Acceptance and Use of Technology

## Data Availability

The datasets generated or analyzed during this study are available from the corresponding author (IV) on reasonable request.
