# Supplementary material for: Adoption of Internet of Things in Health Care: Weighted and Meta-Analytical Review of Theoretical Frameworks and Predictors
Source: J Med Internet Res. 2026 Jan 6;28:e64091. doi: 10.2196/64091 (PMC12820542; doi:10.2196/64091)
Supplement: Multimedia Appendix 1 [file jmir_v28i1e64091_app1.docx]

# Multimedia Appendix 1

## Web of Science:

The search strategy incorporated combinations of keywords, and Boolean operators AND and OR were used to optimize results. The Boolean operator NOT was used to exclude duplicate results from the previous query.

Query 1:

(TS=(healthcare) AND (TS=(mobile)) OR TS=("smart") OR TS=("iot") OR TS=("intelligent")) AND (TS=("use*") OR TS=("attitude") OR TS=(UTAUT*) OR TS=(TAM) OR TS=("intention") OR TS=("adopt*"))

Query 2:

(AB=(healthcare wearable device) OR AB=(medical wearable technology) OR AB=("health management") OR AB=("health measurement") OR AB=("smart") OR AB=("internet of things") OR AB=("iot") OR AB=("intelligent")) AND (AB=("health*")) AND (TS=("intention to adopt") OR TS=("behavioral intention") OR TS=("behavioural intention") OR TI=("acceptance") OR TI=("adopt") OR TI=("adoption") OR TI=("using") OR TI=("use") OR TI=("usage") OR TS=("intention to use") OR TS=("Structural Equation") OR TS=("SEM") OR TS=("UTAUT2") OR TS=("UTAUT") OR TS=("TAM") OR TS=("PLS-SEM")) NOT (TI=(systematic) OR TI=(literature review) OR TI=("post-adoption") OR TI=(diet) OR TI=(nutrition) OR TI=("meditation") OR TI=("contact tracing app") OR TI=("fitness app") OR TI=("eletronic health record") OR TI=("ehr") OR TI=("telehealth") OR TI=("telemedicine") OR TI=("mindfulness app")) NOT #1

We also used Web of Science filters to refine the results by article or early access and by citation topic: “technology acceptance model”, “IoT and edge computing”, “regression techniques”, “smart home automation”, and “health technology assessment.” Most of the papers included in the analysis came from this database.

## PubMed

In PubMed, we referred to controlled vocabulary terms, such as the MeSH terms such as “attitude to computers”, “medical informatics”, “technology assessment, biomedical”, “health behavior”, and “adoption”.

Query 1:

("attitude to computers"[MeSH Terms] OR "medical informatics"[MeSH Terms] OR "technology assessment, biomedical"[MeSH Terms] OR "health behavior"[MeSH Terms] OR "adoption"[MeSH Terms] OR "IoT"[Title/Abstract] OR "internet of things"[Title/Abstract] OR "smart"[Title/Abstract] OR "wearable device"[Title/Abstract] OR "structural equation"[Title/Abstract]) AND ((("adopt"[All Fields] OR "adoptability"[All Fields] OR "adoptable"[All Fields] OR "adopted"[All Fields] OR "adopter"[All Fields] OR "adopters"[All Fields] OR "adopting"[All Fields] OR "adoption"[MeSH Terms] OR "adoption"[All Fields] OR "adoptions"[All Fields] OR "adopts"[All Fields]) AND "use intention"[Title/Abstract]) OR (("usage"[All Fields] OR "usages"[All Fields]) AND "behavior attitude"[Title/Abstract]))

Query 2:

("attitude to computers"[MeSH Terms] OR "medical informatics"[MeSH Terms] OR "technology assessment, biomedical"[MeSH Terms] OR "health behavior"[MeSH Terms] OR "IoT"[Title/Abstract] OR "internet of things"[Title/Abstract] OR "smart"[Title/Abstract] OR "wearable device"[Title/Abstract] OR "structural equation"[Title/Abstract]) AND ((("adopt"[All Fields] OR "adoptability"[All Fields] OR "adoptable"[All Fields] OR "adopted"[All Fields] OR "adopter"[All Fields] OR "adopters"[All Fields] OR "adopting"[All Fields] OR "adoption"[MeSH Terms] OR "adoption"[All Fields] OR "adoptions"[All Fields] OR "adopts"[All Fields]) AND "use intention"[Title/Abstract]) OR (("usage"[All Fields] OR "usages"[All Fields]) AND "behavior attitude"[Title/Abstract]) OR "adoption"[MeSH Terms])

Query 3:

("healthcare"[Title/Abstract] OR "health"[Title/Abstract] OR "medical"[Title/Abstract] OR "clinical"[Title/Abstract] OR "diagnostic"[Title/Abstract] OR "patient"[Title/Abstract] OR "clinician"[Title/Abstract] OR "doctor"[Title/Abstract] OR "diagnostic"[Title/Abstract] OR "treatment"[Title/Abstract] OR "health management"[Title/Abstract] OR "health measurement"[Title/Abstract] OR "technology assessment, biomedical"[MeSH Terms] OR "medical informatics"[MeSH Terms]) AND ("IoT"[Title/Abstract] OR "internet of things"[Title/Abstract] OR "smart"[Title/Abstract] OR "AI-powered"[Title/Abstract] OR "AI-enabled"[Title/Abstract] OR "intelligent"[Title/Abstract] OR "wearable"[Title/Abstract]) AND ("adoption intention"[Title/Abstract] OR "attitude to computers"[MeSH Terms] OR "health behavior"[MeSH Terms] OR "usage behavior"[Title/Abstract] OR "usage intention"[Title/Abstract] OR "intention to adopt"[Title/Abstract] OR "intention to use"[Title/Abstract]) AND ("structural equation"[Title/Abstract] OR "partial least"[Title/Abstract] OR "path analysis"[Title/Abstract] OR "path coefficients"[Title/Abstract] OR "regression coefficients"[Title/Abstract] OR "PLS-SEM"[Title/Abstract] OR "SEM"[Title/Abstract] OR "multiple regression"[Title/Abstract])

## Paper eligibility and data extraction

The papers were accessed for eligibility in Excel. The following are two examples illustrating why a paper would be excluded. For instance, paper [1] was excluded due to the target variable “continued use” not being under study in our review, and the paper [2] due to the methodology used for presenting the results (odds ratio).

For all the studies included in the review, the following aspects, detailed in Table S1, were extracted.

Table S1. Aspects extracted and description.

| **Aspect** | **Description** |
| --- | --- |
| Study Characteristics | Author(s) full names, year of publication, the full title of the article, Digital Object Identifier (DOI), keywords, and abstract. |
| Quality assessment | The name of the journal and the quality ranking of the source according to Scimago. |
| Methodology | The theoretical framework(s) used in the study, the data collection method used, the sample size, details about the sample, and the country of data collection. |
| IoT Technology | The type of technology studied, and the specific device(s) involved in the study (e.g., smart sensors, wearable devices). |
| Effect Measures | Standardized beta coefficients of the relationships between the predictors or explanatory variables and the outcome variables measured in the study and indication of whether the reported effect was statistically significant (S) or not significant (NS). |

## Variable merging

Some variables, representing the same idea based on the definition and measurement items, were merged. Table S2 presents 2 examples.

Table S2: Examples of variables merging

| **Reference** | **Variables** | **Variables merged** |
| --- | --- | --- |
| [3] | Health improvement expectancy | Performance expectancy |
| [4] | Perceived usefulness |  |
| [5] | Performance expectancy |  |
| [6] | Relative advantage |  |
| [7] | Health consciousness | Health consciousness |
| [8] | Health concerns |  |
| [9] | Health interest |  |
| [[10]](http://dx.doi.org/10.3389/fpubh.2022.931557) | Health motivation |  |

## Final list of papers for analysis

All the 115 datasets selected for our analysis are represented in Table S3.

Table S3. The studies included in the analysis, along with the respective references, journal title, quartile, methodology, type of technology, sample size, and country.

| **#** | **Source** | **Year** | **Journal** | **Q** | **Method** | **Technology** | **Sample Size** | **Country** |
| --- | --- | --- | --- | --- | --- | --- | --- | --- |
| 1 | [11] | 2025 | Journal of medical internet research | Q1 | PLS-SEM | Intelligent clinical decision support systems (CDSS) | 247 | China |
| 2 | [12] | 2025 | JMIR aging | Q1 | SEM | Smart health wearable | 306 | Singapore |
| 3 | [13] | 2025 | BMC health services research | Q1 | SEM | AI-enabled technologies for diagnostic | 392 | Germany and USA |
| 4 | [14] | 2025 | Behavioral sciences | Q1 | PLS-SEM | Smart mobile healthcare | 372 | China |
| 5 | [15] | 2025 | Experimental gerontology | Q1 | SEM | Wearable for aging-in-place and patients with chronic diseases | 668 | China |
| 6 | [16] | 2025 | Healthcare | Q2 | PLS-SEM | Artificial intelligence clinical decision support systems (AI-cdsss) | 440 | Romania |
| 7 | [17] | 2025 | Scientific reports | Q1 | CB-SEM | Intelligent care systems | 386 | China |
| 8 | [18] | 2025 | Scientific reports | Q1 | Multivariate regression | Iot-enabled healthcare devices | 772 | Indonesia |
| 9 | [19] | 2025 | JMIR formative research | Q2 | SEM | Smart glasses in healthcare | 450 | USA |
| 10 | [20] | 2025 | Healthcare technology letters | Q2 | PLS-SEM | Digital health tools | 280 | Nepal |
| 11 | [21] | 2025 | Applied nursing research | Q1 | Multiple regression | Iot-based smart mattresses | 159 | South Korea |
| 12 | [22] | 2025 | Bmc public health | Q1 | PLS-SEM | Digital Health consultancy mhealth apps for continuous monitoring and predicting Cardiac health | 495 | Pakistan |
| 13 | [23] | 2025 | UNIVERSAL ACCESS IN THE INFORMATION SOCIETY | Q2 | PLS-SEM | Smart health services for retirement communities | 1292 | China |
| 14 | [24] | 2024 | Sensors | Q1 | PLS-SEM | Iot healthcare devices | 1158 | South Korea |
| 15 | [25] | 2024 | Plos one | Q1 | PLS-SEM | Smart home systems for elderly care | 475 | China |
| 16 | [26] | 2024 | Plos one | Q1 | PLS-SEM | Wearable biofeedback and neurofeedback devices | 106 | United Arab Emirates |
| 17 | [27] | 2024 | Journal of advanced nursing | Q1 | PLS-SEM | AI-assisted care technology | 283 | Taiwan |
| 18 | [28] | 2024 | Digital health | Q2 | PLS-SEM | IoT-enabled wearable healthcare devices (WHDs) | 476 | China |
| 19 | [29] | 2024 | Journal of information & knowledge management | Q2 | PLS-SEM | Smart health applications | 259 | Oman |
| 20 | [30] | 2024 | Health informatics journal | Q2 | Multiple regression | Mobile personal health assistant application | 81 | Taiwan |
| 21 | [31] | 2024 | Buildings | Q1 | PLS-SEM | Smart homes for elderly care | 387 | China |
| 22 | [32] | 2024 | Information | Q2 | SEM | Mobile health | 314 | Pakistan |
| 23 | [33] | 2024 | International journal of pharmaceutical and healthcare marketing | Q3 | Hierarchical regression | Healthcare applications | 238 | India |
| 24 | [34] | 2024 | Frontiers in psychology | Q2 | SEM | Health management application | 624 | China |
| 25 | [35] | 2024 | Frontiers in public health | Q1 | SEM | Smart healthcare technology | 389 | South Korea |
| 26 | [36] | 2024 | Journal of building engineering | Q1 | PLS-SEM | Smart iot applications for healthcare | 200 | Jordan |
| 27 | [37] | 2024 | Frontiers in psychology | Q2 | PLS-SEM | Smart home medical devices | 397 | China |
| 28 | [38] | 2024 | International journal of pharmaceutical and healthcare marketing | Q3 | SEM | Smart wearable technology | 243 | Turkey |
| 29 | [39] | 2024 | IEEE access | Q1 | PLS-SEM | Iot healthcare rehabilitation systems | 158 | China |
| 30 | [40] | 2024 | Plos one | Q1 | SEM | Smart wearable device | 469 | China |
| 31 | [41] | 2023 | JMIR human factors | Q1 | SEM | Artificial intelligence–enabled mental health feedback tools | 206 | Germany, United States, UK, Canada |
| 32 | [42] | 2023 | Kybernetes | Q2 | PLS-SEM | Wearable healthcare technology | 300 | Turkey |
| 33 | [43] | 2023 | BMC geriatrics | Q1 | PLS-SEM | Mobile health applications | 201 | China |
| 34 | [44] | 2023 | International journal of innovation and technology management | Q3 | SEM | IoT healthcare technology | 341 | Iraq |
| 35 | [45] | 2023 | Journal of computer information systems | Q1 | PLS-SEM | IoT wearable devices | 407 | USA |
| 36 | [46] | 2023 | IEEE transactions on engineering management | Q1 | PLS-SEM | Online healthcare application | 292 | China |
| 37 | [47] | 2023 | Journal of global marketing | Q2 | PLS-SEM | Wearable healthcare technologies | 400 | India |
| 38 | [4] | 2023 | Benchmarking-an international journal | Q1 | PLS-SEM | Smart healthcare technology | 913 | Bangladesh |
| 39 | [48] | 2023 | Information development | Q2 | PLS-SEM | IoT-based healthcare systems | 300 | Bangladesh |
| 40 | [49] | 2023 | Research journal of textile and apparel | Q3 | SEM | Smart healthcare wearables | 240 | USA |
| 41 | [50] | 2023 | Clinical epidemiology and global health | Q2 | PLS-SEM | Healthcare wearable devices | 139 | India |
| 42 | [51] | 2023 | Administrative sciences | Q2 | PLS-SEM | Smart health watches | 170 | South Korea |
| 43 | [52] | 2023 | Frontiers in public health | Q1 | PLS-SEM | Smart wearable devices | 357 | China |
| 44 | [53] | 2023 | JMIR aging | Q1 | SEM | Smart IoT app | 477 | South Korea |
| 45 | [54] | 2022 | Journal of multidisciplinary healthcare | Q1 | PLS-SEM | Mobile health applications | 450 | China |
| 46 | [55] | 2022 | FRONTIERS IN PUBLIC HEALTH | Q1 | SEM | Smart wearable activity trackers | 725 | China |
| 47 | [56] | 2022 | International journal of human-computer studies | Q1 | SEM | AI-powered smart healthcare | 322 | Worldwide |
| 48 | [57] | 2022 | International journal of environmental research and public health | Q2 | PLS-SEM | Health mobile apps | 176 | Saudi Arabia |
| 49 | [58] | 2022 | Frontiers in public health | Q1 | PLS-SEM | Mobile health applications | 645 | Ghana |
| 50 | [59] | 2022 | Journal of research in interactive marketing | Q1 | PLS-SEM | Smart wearable devices | 250 | China |
| 51 | [60] | 2022 | Plos one | Q1 | PLS-SEM | Wearable medical devices (WMDs) | 1160 | China |
| 52 | [61] | 2022 | Plos one | Q1 | PLS-SEM | Smart healthcare | 486 | Kingdom of Saudi Arabia |
| 53 | [62] | 2022 | Behavioral sciences | Q2 | Multiple regression | Smart health wearable devices | 166 | Taiwan |
| 54 | [63] | 2022 | Sensors | Q1 | SEM | IoT-based health management tool | 243 | China |
| 55 | [64] | 2022 | Technological forecasting and social change | Q1 | PLS-SEM | IoT-enabled healthcare application | 183 | Oman |
| 56 | [65] | 2022 | Computers in human behavior | Q1 | PLS-SEM | Smart healthcare services | 769 | China |
| 57 | [66] | 2022 | Annals of translational medicine | Q1 | SEM | Wearable intelligent medical devices (WIMDs) | 2192 | China |
| 58 | [67] | 2022 | International journal of environmental research and public health | Q2 | PLS-SEM | Smart home health care services | 487 | South Korea |
| 59 | [68] | 2022 | Technology in society | Q1 | PLS-SEM | Healthcare wearable devices | 534 | India |
| 60 | [3] | 2022 | Frontiers in public health | Q1 | PLS-SEM | Medical wearable devices (MWDs) | 304 | China |
| 61 | [69] | 2022 | Healthcare | Q2 | PLS-SEM | Healthcare wearable devices | 320 | Ghana |
| 62 | [7] | 2022 | Technology in society | Q1 | PLS-SEM | Healthcare wearable devices | 385 | Pakistan |
| 63 | [70] | 2022 | Frontiers in psychology | Q2 | SEM | Mobile health services | 386 | China |
| 64 | [8] | 2022 | Digital health | Q2 | Logistic regression | Wearable healthcare devices | 1063 | South Korea |
| 65 | [8] | 2022 | Digital health | Q2 | Logistic regression | Wearable healthcare devices | 3035 | USA |
| 66 | [71] | 2022 | Telematics and informatics | Q1 | SEM | Mobile healthcare devices | 1294 | South Korea |
| 67 | [72] | 2022 | Technological forecasting and social change | Q1 | SEM | Medical wearable technology | 335 | Taiwan |
| 68 | [73] | 2022 | International journal of environmental research and public health | Q2 | PLS-SEM | Mobile health | 233 | Japan |
| 69 | [74] | 2021 | Risk management and healthcare policy | Q2 | SEM | Pre-exposure prophylaxis intelligent reminder system | 111 | China |
| 70 | [75] | 2021 | Technological forecasting and social change | Q1 | PLS-SEM | IoT healthcare devices | 181 | France |
| 71 | [75] | 2021 | Technological forecasting and social change | Q1 | PLS-SEM | IoT healthcare devices | 267 | France |
| 72 | [76] | 2021 | Informatica-an international journal of computing and informatics | Q2 | SEM | Consumer-oriented health information technologies | 450 | Malaysia |
| 73 | [77] | 2021 | International journal of healthcare information systems and informatics | Q3 | PLS-SEM | Blockchain and IoT technologies in healthcare | 372 | India |
| 74 | [78] | 2021 | Family & consumer sciences research journal | Q1 | SEM | Smart clothing | 376 | USA |
| 75 | [79] | 2021 | Healthcare | Q2 | PLS-SEM | Smart health management | 180 | Taiwan |
| 76 | [80] | 2021 | Future Internet | Q2 | PLS-SEM | Smart watch for medical purposes | 325 | United Arab Emirates |
| 77 | [81] | 2021 | International journal of healthcare information systems and informatics | Q3 | PLS-SEM | IoT medical devices | 153 | India |
| 78 | [82] | 2021 | Journal of global information management | Q2 | PLS-SEM | Smart wearable healthcare devices | 473 | Kingdom of Saudi Arabia |
| 79 | [83] | 2021 | Telematics and Informatics | Q1 | SEM | Wearable healthcare technology | 268 | Taiwan |
| 80 | [84] | 2020 | HEALTH CARE MANAGEMENT SCIENCE | Q1 | SEM | Smart healthcare application for cardiovascular prevention | 212 | Italy |
| 81 | [85] | 2020 | International journal of information management | Q1 | PLS-SEM | Smart wearable healthcare devices | 280 | USA |
| 82 | [86] | 2020 | International journal of pervasive computing and communications | Q2 | SEM | IoT healthcare devices | 124 | Iraq |
| 83 | [87] | 2020 | Plos one | Q1 | PLS-SEM | Smart clothing system | 81 | China |
| 84 | [88] | 2020 | Technological Forecasting and Social Change | Q1 | PLS-SEM | Smart wearable healthcare devices | 273 | India |
| 85 | [89] | 2020 | Sustainability | Q2 | PLS-SEM | Wearable health monitoring technology | 256 | Kingdom of Saudi Arabia |
| 86 | [90] | 2020 | Journal of medical internet research | Q1 | PLS-SEM | Healthcare wearable devices | 201 | China |
| 87 | [90] | 2020 | Journal of medical internet research | Q1 | PLS-SEM | Healthcare wearable devices | 110 | Switzerland |
| 88 | [91] | 2020 | Service business | Q1 | SEM | Healthcare wearable devices | 129 | South Korea |
| 89 | [91] | 2020 | Service business | Q1 | SEM | Healthcare wearable devices | 159 | South Korea |
| 90 | [92] | 2020 | International Journal of Medical Informatics | Q1 | PLS-SEM | Healthcare wearable devices | 406 | China |
| 91 | [93] | 2020 | Technological Forecasting and Social Change | Q1 | SEM | Wearable healthcare technology | 325 | China |
| 92 | [94] | 2020 | Telemedicine journal and e-Health | Q1 | SEM | Smart management app | 458 | Taiwan |
| 93 | [95] | 2020 | Engineering technology & applied science research | Q2 | PLS-SEM | IoT-enabled healthcare | 407 | Kingdom of Saudi Arabia |
| 94 | [96] | 2020 | International journal of production research | Q1 | PLS-SEM | AI-powered smart healthcare | 345 | China |
| 95 | [96] | 2020 | International journal of production research | Q1 | PLS-SEM | AI-powered smart healthcare | 139 | China |
| 96 | [97] | 2020 | Canadian journal of administrative sciences | Q2 | PLS-SEM | Wearable technology devices | 277 | USA |
| 97 | [98] | 2019 | Health psychology research | Q2 | PLS-SEM | Healthcare wearable technology | 171 | China |
| 98 | [99] | 2019 | International journal of health care quality assurance | Q3 | PLS-SEM | Connected healthcare  technologies | 213 | Switzerland |
| 99 | [32] | 2019 | Jmir mhealth and uhealth | Q1 | PLS-SEM | Mobile health services | 395 | China |
| 100 | [100] | 2019 | Journal of innovation economics & management | Q2 | PLS-SEM | Iot-enabled healthcare | 67 | France |
| 101 | [9] | 2019 | Mobile information systems | Q3 | SEM | Wearable healthcare devices | 178 | Malaysia |
| 102 | [101] | 2019 | Applied ergonomics | Q1 | SEM | Smart wearable systems | 146 | China |
| 103 | [102] | 2019 | International Journal of Environmental Research and Public Health | Q2 | PLS-SEM | Smart Wearable healthcare technology | 237 | China |
| 104 | [103] | 2018 | Frontiers of engineering management | Q2 | Path analysis | Smart wearable in healthcare | 71 | Turkey |
| 105 | [104] | 2018 | Journal of enabling technologies | Q3 | PLS-SEM | IoT-based Healthcare wearables | 815 | India |
| 106 | [105] | 2017 | Kybernetes | Q2 | PLS-SEM | IoT products in healthcare | 426 | Turkey |
| 107 | [106] | 2017 | Automation in Construction | Q1 | Multiple regression | IoT wearable technology | 120 | USA |
| 108 | [107] | 2017 | International journal of medical informatics | Q1 | PLS-SEM | Healthcare wearable technology | 197 | China |
| 109 | [107] | 2017 | International journal of medical informatics | Q1 | PLS-SEM | Healthcare wearable technology | 239 | China |
| 110 | [108] | 2017 | Computers in Human Behavior | Q1 | SEM | Healthcare wearable device | 260 | USA |
| 111 | [109] | 2016 | International journal of medical informatics | Q1 | SEM | Healthcare wearable devices | 333 | China |
| 112 | [110] | 2016 | Information technology & people | Q1 | SEM | Wearable healthcare devices | 877 | South Korea |
| 113 | [111] | 2015 | Industrial management & data systems | Q1 | PLS-SEM | Healthcare wearable devices | 462 | China |
| 114 | [112] | 2014 | Telemedicine and e-Health | Q1 | SEM | Wearable health technology | 44 | South Korea |
| 115 | [113] | 2011 | Social behavior and personality | Q3 | SEM | IoT smart healthcare | 125 | Taiwan |

## Bibliography

[1] D. Honglin, Z. Jianghua, and C. Hui, “Quality factors affecting the continued use of mobile health apps in ethnic minority regions of Southwest China using PLS-SEM and ANN,” *Sci Rep*, vol. 14, no. 1, p. 25469, Oct. 2024, doi: 10.1038/s41598-024-75410-4.

[2] R. Chandrasekaran, V. Katthula, and E. Moustakas, “Too old for technology? Use of wearable healthcare devices by older adults and their willingness to share health data with providers,” *Health Informatics J*, vol. 27, no. 4, p. 14604582211058073, Oct. 2021, doi: 10.1177/14604582211058073.

[3] Z. Xinyan, A. A. Mamun, M. H. Ali, L. Siyu, Q. Yang, and N. Hayat, “Modeling the adoption of medical wearable devices among the senior adults: Using hybrid SEM-neural network approach,” *Front. Public Health*, vol. 10, Oct. 2022, doi: 10.3389/fpubh.2022.1016065.

[4] M. O. Gani, M. S. Rahman, S. Bag, and Md. P. Mia, “Examining behavioural intention of using smart health care technology among females: dynamics of social influence and perceived usefulness,” *Benchmarking: An International Journal*, vol. 31, no. 2, pp. 330–352, Jan. 2023, doi: 10.1108/BIJ-09-2022-0585.

[5] J. H. Koo, Y. H. Park, and D. R. Kang, “Factors predicting older people’s acceptance of a personalized health care service app and the effect of chronic disease: Cross-sectional questionnaire study,” *JMIR Aging*, vol. 6, no. 1, p. e41429, Jun. 2023, doi: 10.2196/41429.

[6] S. Bin Naeem, M. Azam, M. N. Kamel Boulos, and R. Bhatti, “Leveraging the TOE Framework: Examining the Potential of Mobile Health (mHealth) to Mitigate Health Inequalities,” *Information*, vol. 15, no. 4, Art. no. 4, Apr. 2024, doi: 10.3390/info15040176.

[7] N. Hayat, A. A. Salameh, H. A. Malik, and M. R. Yaacob, “Exploring the adoption of wearable healthcare devices among the Pakistani adults with dual analysis techniques,” *Technology in Society*, vol. 70, p. 102015, Aug. 2022, doi: 10.1016/j.techsoc.2022.102015.

[8] J.-Y. Rha *et al.*, “What drives the use of wearable healthcare devices? A cross-country comparison between the US and Korea,” *DIGITAL HEALTH*, vol. 8, p. 20552076221120319, Jan. 2022, doi: 10.1177/20552076221120319.

[9] S. Asadi, R. Abdullah, M. Safaei, and S. Nazir, “An Integrated SEM-Neural Network Approach for Predicting Determinants of Adoption of Wearable Healthcare Devices,” *Mobile Information Systems*, vol. 2019, p. e8026042, Feb. 2019, doi: 10.1155/2019/8026042.

[10] N. Hayat, N. R. Zainol, A. A. Salameh, A. Al Mamun, Q. Yang, and M. F. Md Salleh, “How health motivation moderates the effect of intention and usage of wearable medical devices? An empirical study in Malaysia,” *Front. Public Health*, vol. 10, Aug. 2022, doi: 10.3389/fpubh.2022.931557.

[11] R. Zheng *et al.*, “Investigating Clinicians’ Intentions and Influencing Factors for Using an Intelligence-Enabled Diagnostic Clinical Decision Support System in Health Care Systems: Cross-Sectional Survey,” *Journal of Medical Internet Research*, vol. 27, no. 1, p. e62732, Apr. 2025, doi: 10.2196/62732.

[12] H. Kang, T. Yang, N. Banu, S. W. T. Ng, and J. K. Lee, “Exploring Smart Health Wearable Adoption Among Singaporean Older Adults Based on Self-Determination Theory: Web-Based Survey Study,” *JMIR Aging*, vol. 8, no. 1, p. e69008, Mar. 2025, doi: 10.2196/69008.

[13] J. Cecil, A.-K. Kleine, E. Lermer, and S. Gaube, “Mental health practitioners’ perceptions and adoption intentions of AI-enabled technologies: an international mixed-methods study,” *BMC Health Services Research*, vol. 25, no. 1, p. 556, Apr. 2025, doi: 10.1186/s12913-025-12715-8.

[14] J. Luo, K. Zhang, Q. Huang, S. Jiang, and Y. Pan, “From Acceptance to Dependence: Exploring Influences of Smart Healthcare on Continuous Use Intention of Mobile Health Services Among Older Adults with Chronic Illnesses in China,” *Behavioral Sciences*, vol. 15, no. 1, Art. no. 1, Jan. 2025, doi: 10.3390/bs15010019.

[15] Z. Li, P. Chen, and X. Sun, “Influence of personality on wearable activity trackers use among Chinese patients over 50 with type 2 diabetes mellitus: A technology acceptance model perspective,” *Experimental Gerontology*, vol. 208, p. 112813, Sep. 2025, doi: 10.1016/j.exger.2025.112813.

[16] Șerban A. Marinescu, I. Oncioiu, and A.-I. Ghibanu, “The Digital Transformation of Healthcare Through Intelligent Technologies: A Path Dependence-Augmented–Unified Theory of Acceptance and Use of Technology Model for Clinical Decision Support Systems,” *Healthcare*, vol. 13, no. 11, Art. no. 11, Jan. 2025, doi: 10.3390/healthcare13111222.

[17] Z. Wang, Y. Wang, Y. Zeng, J. Su, and Z. Li, “An investigation into the acceptance of intelligent care systems: an extended technology acceptance model (TAM),” *Sci Rep*, vol. 15, no. 1, p. 17912, May 2025, doi: 10.1038/s41598-025-02746-w.

[18] C. A. N. Malarvizhi, A. Al Mamun, M. N. H. Reza, and M. Yang, “Seniors’ attitudes and intention toward IoT enabled healthcare devices in emerging economies,” *Sci Rep*, vol. 15, no. 1, p. 13320, Apr. 2025, doi: 10.1038/s41598-025-97507-0.

[19] N. Zuidhof, O. Peters, P.-P. Verbeek, and S. B. Allouch, “Social Acceptance of Smart Glasses in Health Care: Model Evaluation Study of Anticipated Adoption and Social Interaction,” *JMIR Formative Research*, vol. 9, no. 1, p. e49610, Feb. 2025, doi: 10.2196/49610.

[20] S. M. Timsina and U. Bhattarai, “Identifying factors shaping the behavioural intention of Nepalese youths to adopt digital health tools,” *Healthcare Technology Letters*, vol. 12, no. 1, p. e70005, 2025, doi: 10.1049/htl2.70005.

[21] H. Choi and S. H. Tak, “Family caregivers’ perception of pressure ulcer prevention devices and equipment for patients with cerebrovascular and spinal disease,” *Applied Nursing Research*, vol. 81, p. 151893, Feb. 2025, doi: 10.1016/j.apnr.2024.151893.

[22] S. H. Raza *et al.*, “Catenation between mHealth application advertisements and cardiovascular diseases: moderation of artificial intelligence (AI)-enabled internet of things, digital divide, and individual trust,” *BMC Public Health*, vol. 25, no. 1, p. 1064, Mar. 2025, doi: 10.1186/s12889-025-22082-y.

[23] Y. Li, Y. Ma, Y. Wang, and W. Hong, “The Adoption of smart health services by older adults in retirement communities: analysis with the technology acceptance model (TAM),” *Univ Access Inf Soc*, vol. 24, no. 2, pp. 1105–1121, Jun. 2025, doi: 10.1007/s10209-024-01125-y.

[24] S. Kim, Y. Zhong, J. Wang, and H.-S. Kim, “Exploring Technology Acceptance of Healthcare Devices: The Moderating Role of Device Type and Generation,” *Sensors*, vol. 24, no. 24, Art. no. 24, Jan. 2024, doi: 10.3390/s24247921.

[25] Y. Wang, N. M. Sani, Y. Hua, Q. Jiang, and L. Zhao, “Factors affecting the elderly’s behavioral intention toward smart home systems: A cross-sectional study from China’s eastern coast,” *PLOS ONE*, vol. 19, no. 12, p. e0311280, Dec. 2024, doi: 10.1371/journal.pone.0311280.

[26] S. B. Dias, H. F. Jelinek, and L. J. Hadjileontiadis, “Wearable neurofeedback acceptance model for students’ stress and anxiety management in academic settings,” *PLOS ONE*, vol. 19, no. 10, p. e0304932, Oct. 2024, doi: 10.1371/journal.pone.0304932.

[27] C.-H. Chen and W.-I. Lee, “Exploring Nurses’ Behavioural Intention to Adopt AI Technology: The Perspectives of Social Influence, Perceived Job Stress and Human–Machine Trust,” *Journal of Advanced Nursing*, vol. 81, no. 7, pp. 3739–3752, 2025, doi: 10.1111/jan.16495.

[28] Q. Yang, A. Al Mamun, M. Wu, and F. Naznen, “Strengthening health monitoring: Intention and adoption of Internet of Things-enabled wearable healthcare devices,” *DIGITAL HEALTH*, vol. 10, p. 20552076241279199, Sep. 2024, doi: 10.1177/20552076241279199.

[29] A. Abushakra, D. Nikbin, and L. Anaya, “Navigating Patients’ Intentions in Smart Health: Insights from Novel Factors,” *J. Info. Know. Mgmt.*, p. 2450095, Sep. 2024, doi: 10.1142/S0219649224500953.

[30] M.-C. Kuo, C.-F. Liou, J.-H. Lin, C.-F. Huang, and L.-C. Weng, “Barriers to mobile personal health assistant in patients living with diabetes,” *Health Informatics J*, vol. 30, no. 4, p. 14604582241291522, Oct. 2024, doi: 10.1177/14604582241291522.

[31] Y. Wang, N. M. Sani, B. Shu, Q. Jiang, and H. Lu, “Investigating the Behavioral Intention of Smart Home Systems among Older People in Linyi City,” *Buildings*, vol. 14, no. 10, Art. no. 10, Oct. 2024, doi: 10.3390/buildings14103145.

[32] F. Meng, X. Guo, Z. Peng, K.-H. Lai, and X. Zhao, “Investigating the Adoption of Mobile Health Services by Elderly Users: Trust Transfer Model and Survey Study,” *JMIR mHealth and uHealth*, vol. 7, no. 1, p. e12269, Jan. 2019, doi: 10.2196/12269.

[33] O. J. Gupta, M. K. Srivastava, P. Darda, S. Yadav, and V. Mishra, “How consumer trust affects the adoption of e-healthcare products on mobile apps: an analysis of perceived usefulness, time, and price,” *International Journal of Pharmaceutical and Healthcare Marketing*, vol. ahead-of-print, no. ahead-of-print, Nov. 2024, doi: 10.1108/IJPHM-03-2023-0027.

[34] L. Wang, Y. Zhang, Z. Li, X. Pang, Y. Zhang, and M. Zou, “Analysis of willingness to use health management APP for female college students: application of UTAUT model based on Fogg theory,” *Front. Psychol.*, vol. 15, Nov. 2024, doi: 10.3389/fpsyg.2024.1466566.

[35] C. Wu and G. G. Lim, “Investigating older adults users’ willingness to adopt wearable devices by integrating the technology acceptance model (UTAUT2) and the Technology Readiness Index theory,” *Front. Public Health*, vol. 12, Sep. 2024, doi: 10.3389/fpubh.2024.1449594.

[36] M. Al-Rawashdeh, P. Keikhosrokiani, B. Belaton, M. Alawida, and A. Zwiri, “Effective factors for the adoption of IoT applications in nursing care: A theoretical framework for smart healthcare,” *Journal of Building Engineering*, vol. 89, p. 109012, Jul. 2024, doi: 10.1016/j.jobe.2024.109012.

[37] S. Wu, B. Cui, and X. Yu, “Willingness to use smart fetal heart rate monitoring devices among pregnant women: an extension of the technology acceptance model,” *Front. Psychol.*, vol. 15, Jul. 2024, doi: 10.3389/fpsyg.2024.1400720.

[38] N. Gündüz, S. Zaim, and Y. Ö. Erzurumlu, “Investigating impact of health belief and trust on technology acceptance in smartwatch usage: Turkish senior adults case,” *International Journal of Pharmaceutical and Healthcare Marketing*, vol. 18, no. 3, pp. 499–520, Apr. 2024, doi: 10.1108/IJPHM-11-2022-0102.

[39] F. Li, D. Tolessa Negera, M. Adnan Zahid Chudhery, Q. Zhao, and L. Gao, “IoT and Motion Recognition-Based Healthcare Rehabilitation Systems (IMRHRS): An Empirical Examination From Physicians’ Perspective Using Stimulus-Organism-Response Theory,” *IEEE Access*, vol. 12, pp. 142863–142882, 2024, doi: 10.1109/ACCESS.2024.3464101.

[40] L. Song, B. Li, H. Wu, C. Wu, and X. Zhang, “Understanding the factors of wearable devices among the patients with thyroid cancer: A modified UTAUT2 model,” *PLOS ONE*, vol. 19, no. 7, p. e0305944, Jul. 2024, doi: 10.1371/journal.pone.0305944.

[41] A.-K. Kleine, E. Kokje, E. Lermer, and S. Gaube, “Attitudes Toward the Adoption of 2 Artificial Intelligence–Enabled Mental Health Tools Among Prospective Psychotherapists: Cross-sectional Study,” *JMIR Human Factors*, vol. 10, no. 1, p. e46859, Jul. 2023, doi: 10.2196/46859.

[42] K. Toker, M. Afacan Fındıklı, Z. İ. Gözübol, and A. Görener, “To be a cyborg or not: exploring the mechanisms between digital literacy and neural implant acceptance,” *Kybernetes*, Art. no. ahead-of-print, Jan. 2023, doi: 10.1108/K-07-2023-1297.

[43] J. Y. W. Liu, G. Sorwar, M. S. Rahman, and M. R. Hoque, “The role of trust and habit in the adoption of mHealth by older adults in Hong Kong: a healthcare technology service acceptance (HTSA) model,” *BMC Geriatrics*, vol. 23, no. 1, p. 73, Feb. 2023, doi: 10.1186/s12877-023-03779-4.

[44] S. Salah and B. A. Alyouzbaky, “Assessment of the Acceptance of Internet of Things Technology in the Health Field Using TAM2: A Survey Study of Medical Staff in Nineveh Governorate,” *Int. J. Innovation Technol. Management*, vol. 20, no. 08, p. 2350055, Dec. 2023, doi: 10.1142/S0219877023500554.

[45] K. Guerra, C. Koh, V. Prybutok, and V. Johnson, “WIoT Adoption Among Young Adults in Healthcare Crises,” *Journal of Computer Information Systems*, vol. 63, no. 6, pp. 1316–1331, Nov. 2023, doi: 10.1080/08874417.2022.2150911.

[46] M. A. Z. Chudhery, S. Safdar, J. Huo, H.-U. Rehman, and R. Rafique, “Proposing and Empirically Investigating a Mobile-Based Outpatient Healthcare Service Delivery Framework Using Stimulus–Organism–Response Theory,” *IEEE Transactions on Engineering Management*, vol. 70, no. 8, pp. 2668–2681, Aug. 2023, doi: 10.1109/TEM.2021.3081571.

[47] S. Singh, “The Moderating Role of Privacy Concerns on Intention to Use Smart Wearable Technologies: An Integrated Model Combining UTAUT2 Theoretical Framework and Privacy Dimensions,” *Journal of Global Marketing*, vol. 36, no. 2, pp. 93–111, Mar. 2023, doi: 10.1080/08911762.2022.2141167.

[48] N. Hasan, Y. Bao, S. J. Miah, and A. Fenton, “Factors influencing the young physicians’ intention to use Internet of Things (IoT) services in healthcare,” *Information Development*, vol. 39, no. 4, pp. 902–919, Nov. 2023, doi: 10.1177/02666669211064114.

[49] Z. Tong, S. Lee, and H. Woo, “The effects of product-brand fit and brand type on wearable smart masks,” *Research Journal of Textile and Apparel*, vol. ahead-of-print, no. ahead-of-print, Jan. 2023, doi: 10.1108/RJTA-05-2023-0054.

[50] S. Misra, R. Adtani, Y. Singh, S. Singh, and D. Thakkar, “Exploring the factors affecting behavioral intention to adopt wearable devices,” *Clinical Epidemiology and Global Health*, vol. 24, p. 101428, Nov. 2023, doi: 10.1016/j.cegh.2023.101428.

[51] K. S. L. T. Zin, S. Kim, H.-S. Kim, and I. F. Feyissa, “A Study on Technology Acceptance of Digital Healthcare among Older Korean Adults Using Extended Tam (Extended Technology Acceptance Model),” *Administrative Sciences*, vol. 13, no. 2, Art. no. 2, Feb. 2023, doi: 10.3390/admsci13020042.

[52] J. Chen, T. Wang, Z. Fang, and H. Wang, “Research on elderly users’ intentions to accept wearable devices based on the improved UTAUT model,” *Front. Public Health*, vol. 10, Jan. 2023, doi: 10.3389/fpubh.2022.1035398.

[53] J. H. Koo, Y. H. Park, and D. R. Kang, “Factors Predicting Older People’s Acceptance of a Personalized Health Care Service App and the Effect of Chronic Disease: Cross-Sectional Questionnaire Study,” *JMIR Aging*, vol. 6, no. 1, p. e41429, Jun. 2023, doi: 10.2196/41429.

[54] Q. Xu, X. Hou, T. Xiao, and W. Zhao, “Factors Affecting Medical Students&rsquo; Continuance Intention to Use Mobile Health Applications,” *JMDH*, vol. 15, pp. 471–484, Mar. 2022, doi: 10.2147/JMDH.S327347.

[55] P. Chen, Y. Shen, Z. Li, X. Sun, X. L. Feng, and E. B. Fisher, “What Factors Predict the Adoption of Type 2 Diabetes Patients to Wearable Activity Trackers—Application of Diffusion of Innovation Theory,” *Front. Public Health*, vol. 9, Jan. 2022, doi: 10.3389/fpubh.2021.773293.

[56] F. M. Calisto, N. Nunes, and J. C. Nascimento, “Modeling adoption of intelligent agents in medical imaging,” *International Journal of Human-Computer Studies*, vol. 168, p. 102922, Dec. 2022, doi: 10.1016/j.ijhcs.2022.102922.

[57] N. S. Alharbi, A. S. AlGhanmi, and M. Fahlevi, “Adoption of Health Mobile Apps during the COVID-19 Lockdown: A Health Belief Model Approach,” *International Journal of Environmental Research and Public Health*, vol. 19, no. 7, Art. no. 7, Jan. 2022, doi: 10.3390/ijerph19074179.

[58] I. K. Mensah, “Understanding the Drivers of Ghanaian Citizens’ Adoption Intentions of Mobile Health Services,” *Front. Public Health*, vol. 10, Jun. 2022, doi: 10.3389/fpubh.2022.906106.

[59] Y. Zhu, Y. Lu, S. Gupta, J. Wang, and P. Hu, “Promoting smart wearable devices in the health-AI market: the role of health consciousness and privacy protection,” *Journal of Research in Interactive Marketing*, vol. 17, no. 2, pp. 257–272, Jan. 2022, doi: 10.1108/JRIM-10-2021-0246.

[60] Q. Yang, A. A. Mamun, N. Hayat, M. F. M. Salleh, G. Jingzu, and N. R. Zainol, “Modelling the mass adoption potential of wearable medical devices,” *PLOS ONE*, vol. 17, no. 6, p. e0269256, Jun. 2022, doi: 10.1371/journal.pone.0269256.

[61] K. Maswadi, N. A. Ghani, and S. Hamid, “Factors influencing the elderly’s behavioural intention to use smart home technologies in Saudi Arabia,” *PLOS ONE*, vol. 17, no. 8, p. e0272525, Aug. 2022, doi: 10.1371/journal.pone.0272525.

[62] M.-Y. Jeng, F.-Y. Pai, and T.-M. Yeh, “Antecedents for Older Adults’ Intention to Use Smart Health Wearable Devices-Technology Anxiety as a Moderator,” *Behavioral Sciences*, vol. 12, no. 4, Art. no. 4, Apr. 2022, doi: 10.3390/bs12040114.

[63] D. Liu, Q. Li, and S. Han, “Using Extended Technology Acceptance Model to Assess the Adopt Intention of a Proposed IoT-Based Health Management Tool,” *Sensors*, vol. 22, no. 16, Art. no. 16, Jan. 2022, doi: 10.3390/s22166092.

[64] M. Alraja, “Frontline healthcare providers’ behavioural intention to Internet of Things (IoT)-enabled healthcare applications: A gender-based, cross-generational study,” *Technological Forecasting and Social Change*, vol. 174, p. 121256, Jan. 2022, doi: 10.1016/j.techfore.2021.121256.

[65] K. Liu and D. Tao, “The roles of trust, personalization, loss of privacy, and anthropomorphism in public acceptance of smart healthcare services,” *Computers in Human Behavior*, vol. 127, p. 107026, Feb. 2022, doi: 10.1016/j.chb.2021.107026.

[66] Z. Yin, J. Yan, S. Fang, D. Wang, and D. Han, “User acceptance of wearable intelligent medical devices through a modified unified theory of acceptance and use of technology,” *Annals of Translational Medicine*, vol. 10, no. 11, Art. no. 11, Jun. 2022, doi: 10.21037/atm-21-5510.

[67] H.-J. Kang, J. Han, and G. H. Kwon, “The Acceptance Behavior of Smart Home Health Care Services in South Korea: An Integrated Model of UTAUT and TTF,” *International Journal of Environmental Research and Public Health*, vol. 19, no. 20, Art. no. 20, Jan. 2022, doi: 10.3390/ijerph192013279.

[68] N. Singh, R. Misra, S. Singh, N. P. Rana, and S. Khorana, “Assessing the factors that influence the adoption of healthcare wearables by the older population using an extended PMT model,” *Technology in Society*, vol. 71, p. 102126, Nov. 2022, doi: 10.1016/j.techsoc.2022.102126.

[69] E. Larnyo *et al.*, “Impact of Actual Use Behavior of Healthcare Wearable Devices on Quality of Life: A Cross-Sectional Survey of People with Dementia and Their Caregivers in Ghana,” *Healthcare*, vol. 10, no. 2, Art. no. 2, Feb. 2022, doi: 10.3390/healthcare10020275.

[70] Y. Liu, X. Lu, G. Zhao, C. Li, and J. Shi, “Adoption of mobile health services using the unified theory of acceptance and use of technology model: Self-efficacy and privacy concerns,” *Front. Psychol.*, vol. 13, Aug. 2022, doi: 10.3389/fpsyg.2022.944976.

[71] H. Ahn and E. Park, “Determinants of consumer acceptance of mobile healthcare devices: An application of the concepts of technology acceptance and coolness,” *Telematics and Informatics*, vol. 70, p. 101810, May 2022, doi: 10.1016/j.tele.2022.101810.

[72] K.-H. Huarng, T. H.-K. Yu, and C. fang Lee, “Adoption model of healthcare wearable devices,” *Technological Forecasting and Social Change*, vol. 174, p. 121286, Jan. 2022, doi: 10.1016/j.techfore.2021.121286.

[73] J. Cao, K. Kurata, Y. Lim, S. Sengoku, and K. Kodama, “Social Acceptance of Mobile Health among Young Adults in Japan: An Extension of the UTAUT Model,” *International Journal of Environmental Research and Public Health*, vol. 19, no. 22, Art. no. 22, Jan. 2022, doi: 10.3390/ijerph192215156.

[74] Y. Ma, X. Zhong, B. Lin, and W. He, “Factors Influencing the Intention of MSM to Use the PrEP Intelligent Reminder System,” *RMHP*, vol. 14, pp. 4739–4748, Nov. 2021, doi: 10.2147/RMHP.S337287.

[75] W. Ben Arfi, I. Ben Nasr, T. Khvatova, and Y. Ben Zaied, “Understanding acceptance of eHealthcare by IoT natives and IoT immigrants: An integrated model of UTAUT, perceived risk, and financial cost,” *Technological Forecasting and Social Change*, vol. 163, p. 120437, Feb. 2021, doi: 10.1016/j.techfore.2020.120437.

[76] M. Sinha, L. Fukey, K. Balasubramanian, M. H. Hanafiah, P. Kunasekaran, and N. A. Ragavan, “Acceptance of Consumer-Oriented Health Information Technologies (CHITs): Integrating Technology Acceptance Model with Perceived Risk,” *Informatica*, vol. 45, no. 6, Art. no. 6, Oct. 2021, doi: 10.31449/inf.v45i6.3484.

[77] S. Kuberkar and T. K. Singhal, “Factors Influencing the Adoption Intention of Blockchain and Internet-of-Things Technologies for Sustainable Blood Bank Management,” *International Journal of Healthcare Information Systems and Informatics (IJHISI)*, vol. 16, no. 4, pp. 1–21, 2021, doi: 10.4018/IJHISI.20211001.oa15.

[78] N. Mahmood and Y.-A. Lee, “Factors Influencing Older Adults’ Acceptance of Health Monitoring Smart Clothing,” *Family and Consumer Sciences Research Journal*, vol. 49, no. 4, pp. 376–392, 2021, doi: 10.1111/fcsr.12404.

[79] W.-C. Chi, W.-C. Cheng, T.-H. Chen, and P.-J. Lin, “Impact of Using the Intelligent Physical Health Measurement System on Active Aging: A Survey in Taiwan,” *Healthcare*, vol. 9, no. 9, Art. no. 9, Sep. 2021, doi: 10.3390/healthcare9091142.

[80] R. S. Al-Maroof, K. Alhumaid, A. Q. Alhamad, A. Aburayya, and S. Salloum, “User Acceptance of Smart Watch for Medical Purposes: An Empirical Study,” *Future Internet*, vol. 13, no. 5, Art. no. 5, May 2021, doi: 10.3390/fi13050127.

[81] R. Jain and V. B. S, “Doctors’ Perceptions on the Use of Internet of Things Medical Devices (IOT-MDs) for Anemic Pregnant Women: A TAM2 Study,” *International Journal of Healthcare Information Systems and Informatics (IJHISI)*, vol. 16, no. 1, pp. 58–80, 2021, doi: 10.4018/IJHISI.2021010104.

[82] I. H. Rehman, A. Ahmad, F. Akhter, and A. Aljarallah, “A Dual-Stage SEM-ANN Analysis to Explore Consumer Adoption of Smart Wearable Healthcare Devices,” *Journal of Global Information Management (JGIM)*, vol. 29, no. 6, pp. 1–30, 2021, doi: 10.4018/JGIM.294123.

[83] T. B. Kim and C.-T. B. Ho, “Validating the moderating role of age in multi-perspective acceptance model of wearable healthcare technology,” *Telematics and Informatics*, vol. 61, p. 101603, Aug. 2021, doi: 10.1016/j.tele.2021.101603.

[84] D. Bettiga, L. Lamberti, and E. Lettieri, “Individuals’ adoption of smart technologies for preventive health care: a structural equation modeling approach,” *Health Care Manag Sci*, vol. 23, no. 2, pp. 203–214, Jun. 2020, doi: 10.1007/s10729-019-09468-2.

[85] S. Farivar, M. Abouzahra, and M. Ghasemaghaei, “Wearable device adoption among older adults: A mixed-methods study,” *International Journal of Information Management*, vol. 55, p. 102209, Dec. 2020, doi: 10.1016/j.ijinfomgt.2020.102209.

[86] A. Alhasan, L. Audah, I. Ibrahim, A. Al-Sharaa, A. S. Al-Ogaili, and J. M. Mohammed, “A case-study to examine doctors’ intentions to use IoT healthcare devices in Iraq during COVID-19 pandemic,” *International Journal of Pervasive Computing and Communications*, vol. 18, no. 5, pp. 527–547, Jan. 2020, doi: 10.1108/IJPCC-10-2020-0175.

[87] T.-H. Tsai, W.-Y. Lin, Y.-S. Chang, P.-C. Chang, and M.-Y. Lee, “Technology anxiety and resistance to change behavioral study of a wearable cardiac warming system using an extended TAM for older adults,” *PLOS ONE*, vol. 15, no. 1, p. e0227270, Jan. 2020, doi: 10.1371/journal.pone.0227270.

[88] A. Papa, M. Mital, P. Pisano, and M. Del Giudice, “E-health and wellbeing monitoring using smart healthcare devices: An empirical investigation,” *Technological Forecasting and Social Change*, vol. 153, p. 119226, Apr. 2020, doi: 10.1016/j.techfore.2018.02.018.

[89] S. S. Binyamin and M. R. Hoque, “Understanding the Drivers of Wearable Health Monitoring Technology: An Extension of the Unified Theory of Acceptance and Use of Technology,” *Sustainability*, vol. 12, no. 22, Art. no. 22, Jan. 2020, doi: 10.3390/su12229605.

[90] D. Y. Meier, P. Barthelmess, W. Sun, and F. Liberatore, “Wearable Technology Acceptance in Health Care Based on National Culture Differences: Cross-Country Analysis Between Chinese and Swiss Consumers,” *Journal of Medical Internet Research*, vol. 22, no. 10, p. e18801, Oct. 2020, doi: 10.2196/18801.

[91] S. M. Lee and D. Lee, “Healthcare wearable devices: an analysis of key factors for continuous use intention,” *Serv Bus*, vol. 14, no. 4, pp. 503–531, Dec. 2020, doi: 10.1007/s11628-020-00428-3.

[92] H. Wang, D. Tao, N. Yu, and X. Qu, “Understanding consumer acceptance of healthcare wearable devices: An integrated model of UTAUT and TTF,” *International Journal of Medical Informatics*, vol. 139, p. 104156, Jul. 2020, doi: 10.1016/j.ijmedinf.2020.104156.

[93] Md. S. Talukder, G. Sorwar, Y. Bao, J. U. Ahmed, and Md. A. S. Palash, “Predicting antecedents of wearable healthcare technology acceptance by elderly: A combined SEM-Neural Network approach,” *Technological Forecasting and Social Change*, vol. 150, p. 119793, Jan. 2020, doi: 10.1016/j.techfore.2019.119793.

[94] C.-Y. Huang and M.-C. Yang, “Empirical Investigation of Factors Influencing Consumer Intention to Use an Artificial Intelligence-Powered Mobile Application for Weight Loss and Health Management,” *Telemedicine and e-Health*, vol. 26, no. 10, pp. 1240–1251, Oct. 2020, doi: 10.1089/tmj.2019.0182.

[95] M. H. Alanazi and B. Soh, “Behavioral Intention to Use IoT Technology in Healthcare Settings,” *Engineering, Technology & Applied Science Research*, vol. 9, no. 5, Art. no. 5, Oct. 2019, doi: 10.48084/etasr.3063.

[96] J. Pan, S. Ding, D. Wu, S. Yang, and J. Yang, “Exploring behavioural intentions toward smart healthcare services among medical practitioners: a technology transfer perspective,” *International Journal of Production Research*, vol. 57, no. 18, pp. 5801–5820, Sep. 2019, doi: 10.1080/00207543.2018.1550272.

[97] K. Sergueeva, N. Shaw, and S. H. (Mark) Lee, “Understanding the barriers and factors associated with consumer adoption of wearable technology devices in managing personal health,” *Canadian Journal of Administrative Sciences / Revue Canadienne des Sciences de l’Administration*, vol. 37, no. 1, pp. 45–60, 2020, doi: 10.1002/cjas.1547.

[98] K. Y. Chau *et al.*, “Smart technology for healthcare: Exploring the antecedents of adoption intention of healthcare wearable technology,” *Health Psychol Res*, vol. 7, no. 1, p. 8099, Sep. 2019, doi: 10.4081/hpr.2019.8099.

[99] R. Etemad-Sajadi and G. G. D. Santos, “Senior citizens’ acceptance of connected health technologies in their homes,” *International Journal of Health Care Quality Assurance*, vol. 32, no. 8, pp. 1162–1174, Oct. 2019, doi: 10.1108/IJHCQA-10-2018-0240.

[100] P. Baudier, C. Ammi, and A. Lecouteux, “Employees’ Acceptance of the Healthcare Internet of Things: A Source of Innovation in Corporate Human Resource Policies,” *Journal of Innovation Economics & Management*, vol. 30, no. 3, pp. 89–111, 2019, doi: 10.3917/jie.pr1.051.

[101] J. Li, Q. Ma, A. HS. Chan, and S. S. Man, “Health monitoring through wearable technologies for older adults: Smart wearables acceptance model,” *Applied Ergonomics*, vol. 75, pp. 162–169, Feb. 2019, doi: 10.1016/j.apergo.2018.10.006.

[102] M. L. Cheung *et al.*, “Examining Consumers’ Adoption of Wearable Healthcare Technology: The Role of Health Attributes,” *International Journal of Environmental Research and Public Health*, vol. 16, no. 13, Art. no. 13, Jan. 2019, doi: 10.3390/ijerph16132257.

[103] N. Basoglu, M. Goken, M. Dabic, D. O. Gungor, and T. U. Daim, “Exploring adoption of augmented reality smart glasses: Applications in the medical industry,” *Front. Eng*, vol. 5, no. 2, Art. no. 2, Jun. 2018, doi: 10.15302/J-FEM-2018056.

[104] B. Sivathanu, “Adoption of internet of things (IOT) based wearables for healthcare of older adults – a behavioural reasoning theory (BRT) approach,” *Journal of Enabling Technologies*, vol. 12, no. 4, pp. 169–185, Jan. 2018, doi: 10.1108/JET-12-2017-0048.

[105] A. Karahoca, D. Karahoca, and M. Aksöz, “Examining intention to adopt to internet of things in healthcare technology products,” *Kybernetes*, vol. 47, no. 4, pp. 742–770, Jan. 2017, doi: 10.1108/K-02-2017-0045.

[106] B. Choi, S. Hwang, and S. Lee, “What drives construction workers’ acceptance of wearable technologies in the workplace?: Indoor localization and wearable health devices for occupational safety and health,” *Automation in Construction*, vol. 84, pp. 31–41, Dec. 2017, doi: 10.1016/j.autcon.2017.08.005.

[107] M. Zhang, M. Luo, R. Nie, and Y. Zhang, “Technical attributes, health attribute, consumer attributes and their roles in adoption intention of healthcare wearable technology,” *International Journal of Medical Informatics*, vol. 108, pp. 97–109, Dec. 2017, doi: 10.1016/j.ijmedinf.2017.09.016.

[108] A. Marakhimov and J. Joo, “Consumer adaptation and infusion of wearable devices for healthcare,” *Computers in Human Behavior*, vol. 76, pp. 135–148, Nov. 2017, doi: 10.1016/j.chb.2017.07.016.

[109] H. Li, J. Wu, Y. Gao, and Y. Shi, “Examining individuals’ adoption of healthcare wearable devices: An empirical study from privacy calculus perspective,” *International Journal of Medical Informatics*, vol. 88, pp. 8–17, Apr. 2016, doi: 10.1016/j.ijmedinf.2015.12.010.

[110] E. Park, K. J. Kim, and S. J. Kwon, “Understanding the emergence of wearable devices as next-generation tools for health communication,” *Information Technology & People*, vol. 29, no. 4, pp. 717–732, Jan. 2016, doi: 10.1108/ITP-04-2015-0096.

[111] Y. Gao, H. Li, and Y. Luo, “An empirical study of wearable technology acceptance in healthcare,” *Industrial Management & Data Systems*, vol. 115, no. 9, pp. 1704–1723, Jan. 2015, doi: 10.1108/IMDS-03-2015-0087.

[112] J. Kim, “Analysis of Health Consumers’ Behavior Using Self-Tracker for Activity, Sleep, and Diet,” *Telemedicine and e-Health*, vol. 20, no. 6, pp. 552–558, Jun. 2014, doi: 10.1089/tmj.2013.0282.

[113] C.-L. Hsu, K. C. Tseng, and Y.-H. Chuang, “Predictors of Future Use of Telehomecare Health Services by Middle-Aged People in Taiwan,” *Social Behavior and Personality: an international journal*, vol. 39, no. 9, pp. 1251–1261, Oct. 2011, doi: 10.2224/sbp.2011.39.9.1251.
